# The impact of restrictions on neonicotinoid and fipronil insecticides on pest management in maize, oilseed rape and sunflower in eight European Union regions†

**DOI:** 10.1002/ps.4715

**Published:** 2017-10-13

**Authors:** Jonas Kathage, Pedro Castañera, José Luis Alonso‐Prados, Manuel Gómez‐Barbero, Emilio Rodríguez‐Cerezo

**Affiliations:** ^1^ European Commission, Joint Research Centre (JRC), Directorate for Sustainable Resources Economics of Agriculture Unit Seville Spain; ^2^ Spanish National Research Council (CSIC) Biological Research Center (CIB) Madrid Spain; ^3^ National Institute for Agricultural and Food Research and Technology (INIA) DTEVPF Madrid Spain

**Keywords:** insecticides, clothianidin, imidacloprid, thiamethoxam, neonicotinoids, fipronil

## Abstract

**BACKGROUND:**

In 2013, the European Commission restricted the use of three neonicotinoids (clothianidin, imidacloprid and thiamethoxam) and the pyrazole fipronil, which are widely used to control early‐season pests. Here, we used original farm survey data to examine the impact of the restrictions on pest management practices in eight regional case studies including maize, oilseed rape and sunflower in seven European Union (EU) countries.

**RESULTS:**

In four case studies, farmers switched to using untreated seeds as no alternative seed treatments were available. In three case studies, farmers switched to using unrestricted neonicotinoid‐ or pyrethroid‐treated seeds. In five case studies, farmers increased the use of soil or foliar treatments, with pyrethroids as the principal insecticide class. Other changes in pest management practices ranged from increased sowing density to more frequent scouting for pests. Many farmers perceived that the time, cost and amount of insecticides required to protect crops increased, along with pest pressure. Alternative seed treatments were mostly perceived as being less effective than the restricted seed treatments.

**CONCLUSION:**

Farmers generally relied on alternative seed treatments or more soil/foliar treatments in the first growing season after the restrictions took effect. Further study is required to assess the effectiveness and sustainability of these alternatives compared with the restricted insecticides. © 2017 The Authors. *Pest Management Science* published by John Wiley & Sons Ltd on behalf of Society of Chemical Industry.

## 1 INTRODUCTION

Neonicotinoids are a group of systemic insecticides that are widely used in agricultural crops. In 2014, neonicotinoids accounted for >25% of global insecticide sales.^1^ About 60% of neonicotinoids are employed for seed and soil treatments against soil‐dwelling arthropods and early‐season leaf‐feeding and sucking insect pests. The neonicotinoids with the largest market are imidacloprid, thiamethoxam and clothianidin, which are widely used for controlling pests that attack seeds and seedlings of maize, oilseed rape (OSR) and sunflower.^2^

In May 2013, the European Commission published Regulation (EU) No 485/2013, establishing certain restrictions on the use of clothianidin, thiamethoxam and imidacloprid, following an assessment of their risk to bees by the European Food Safety Authority (EFSA).^3^, ^4^, ^5^, ^6^ The restrictions were specified for different crops and uses. For maize, OSR and sunflower, the most relevant seed, soil and foliar treatments were banned across the European Union (EU) from 1 December 2013 (only foliar treatments after flowering were still allowed). In addition to these three neonicotinoids, the use of another insecticide approved for seed treatment, fipronil, was restricted by the European Commission in July 2013 under Regulation (EU) No 781/2013 after the EFSA had identified a high acute risk to bees.^7^, ^8^ Since 1 March 2014, fipronil has been prohibited for maize and sunflower, for which it was previously authorised in several countries. Before these EU‐wide restrictions on clothianidin, imidacloprid, thiamethoxam and fipronil (CITF) were approved, a few European countries including France, Germany, Italy and Slovenia had already implemented partial restrictions on neonicotinoid use because of concerns regarding bees.^9^, ^10^, ^11^, ^12^ Meanwhile, several other countries have granted farmers derogations from the EU restrictions for certain CITF products and uses since 2014.

After the EU restrictions were enacted, research on the risks posed by CITF to honey bees and other pollinators has continued and intensified.^9^, ^13^, ^14^, ^15^, ^16^, ^17^, ^18^, ^19^, ^20^, ^21^, ^22^, ^23^, ^24^, ^25^, ^26^, ^27^ However, the study of alternatives to CITF, particularly available insecticides and their agronomic, economic and environmental performance, has received less attention. The effects of the restrictions on pollinators and other non‐target organisms will depend on the adaptations in pest management practices that farmers make in response to the restrictions and in particular on the comparative risk profiles of CITF and the insecticides that farmers may use as substitutes. The effects of the restrictions on farmers in terms of crop protection also depend on the effectiveness and cost of the alternative substances and pest management practices. To the extent that the restrictions may influence agricultural productivity and the crop choices farmers make, pollinators and other non‐target organisms may also face a change in the availability of accessible food sources. In addition, effects on pollinators can also influence productivity in some crops. Restrictions on CITF can thus have direct and indirect effects, which are determined by the substitution of substances and agronomic practices by farmers. Therefore, a necessary step towards understanding the economic and environmental effects of the restrictions is to produce empirical evidence of how farmers have adapted their pest management practices in response to the restrictions.

Some authors have predicted potential agronomic, economic and environmental impacts of a complete or partial restriction of neonicotinoids in the EU.^12^, ^20^, ^28^, ^29^, ^30^, ^31^, ^32^, ^33^, ^34^, ^35^, ^36^, ^37^ These predictions were based on literature reviews and/or data collected from farmers, industry, experts and stakeholders and focused in most cases on the cultivation of OSR in the United Kingdom (UK). The general view is that restrictions on neonicotinoid use for seed treatments would result in an increased number of insecticide applications and/or other changes in pest management practices, for example modifying sowing dates and sowing densities. Several studies also predicted yield and/or economic losses for farmers in some cases but not in others.

More recently, several studies have started to approach the issue by analysing data on pest management practices from growing seasons after the EU restrictions took effect (2014/2015 and 2015/2016). These studies again have focused almost exclusively on OSR cultivation in the UK; for other countries and crops, little or no evidence is available.^36^, ^38^, ^39^, ^40^, ^41^, ^42^, ^43^, ^44^, ^45^, ^46^, ^47^, ^48^, ^49^, ^50^, ^51^, ^52^, ^53^, ^54^ The studies of OSR in the UK, as well as two studies in Germany and Hungary, suggest that the neonicotinoid restrictions have had a significant effect on pest management, mostly in terms of increasing the number of foliar treatments with pyrethroids, and also other adaptations such as modifications of the sowing date or seed rate, with some evidence that this costs farmers more time and money. There are also indications of increased pest pressure and reductions in the area of OSR grown, although the evidence regarding yield effects is less clear. In addition to focusing mostly on OSR in the UK, several of these studies were focused only on a few specific effects (e.g. pest pressure).

The present study was designed as a systematic and wide‐ranging analysis of the changes in pest management practices in response to the EU restrictions on CITF. For this purpose, we looked in detail at eight regional case studies from seven EU countries with original farm survey data, taking into account the most relevant affected crops and growing regions. The surveys have allowed us to study the pest management practices employed by maize growers in Aquitaine (France), Lombardy (Italy) and Aragon (Spain), OSR growers in the Czech Republic, Eastern Germany and the East of England (UK), and sunflower growers in Andalusia (Spain) and the Northern Great Plain (Hungary) before and after the CITF restrictions took effect. We collected data using farm surveys involving a total of 800 farmers (100 per case) who used CITF before the restrictions. Each farmer provided details on insecticide use for two growing seasons (2012/2013 and 2013/2014) before the restrictions came into effect and one growing season afterwards (2014/2015), allowing us to observe the impact of the restrictions. The dataset also contains information about other changes made in response to the restrictions, and farmers' perceptions of various impacts of the restrictions.

## 2 METHODS

Eight case studies, each of which consisted of a crop and a region within a country, were selected based on several criteria, namely (1) the severity of the restrictions for a crop, (2) the estimated adoption rate of CITF in a crop, (3) the production volume of a crop, and (4) geographical balance. Criteria (1) and (2) were applied in order to focus on the cases where the restrictions were most likely to produce a change in pest management. Crops with less severe restrictions or low adoption of CITF were expected to see fewer aggregate impacts; pest management in crops not included in the restrictions or without prior CITF use was not likely to change as a direct response. Criterion (3) was applied because larger production volume in arable crops tends to reflect greater economic importance and a larger cultivated area, both of which are a proxy for the overall magnitude of impacts on farmers. Criterion (4) was applied in order for the study to cover different geographical areas.

The major arable crops in which CITF use was widespread and for which the restrictions were most severe (essentially banning all uses) are maize, OSR and sunflower. For other major crops, including potatoes, sugar beet and wheat, the restrictions were less severe because these crops were considered less attractive to bees. Having chosen maize, OSR and sunflower, we considered the selection of countries and regions, again relying on criteria (1), (2) and (3), as well as criterion (4). Table 1 shows the largest EU producers of the three crops, the authorisation status of products containing CITF before the restrictions, and derogations granted after the restrictions.^3^, ^4^, ^5^, ^7^, ^55^ Countries that had no authorised products containing CITF or that granted extensive derogations were not considered for the survey because the restrictions were less likely to have an effect on farmers there. For maize, France and Italy were selected as they were the leading producers, and Spain was chosen over Romania (extensive derogations), Germany (no authorised products) and Hungary (chosen for sunflower). Italy had had national restrictions on the use of neonicotinoid seed treatments in maize in place since 2008, but soil and foliar treatments with neonicotinoids were still allowed. Italy was also selected to observe medium‐term impacts of restrictions on CITF seed treatments. For OSR, Germany, the UK and the Czech Republic were selected (the leading producer France had no authorised CITF products). For sunflower, France was excluded for not having authorised products and Romania for extensive derogations. Hungary was chosen over Bulgaria because of a larger number of authorised products, and Spain was selected as a Mediterranean country. In the next step, desk research was conducted for criteria (1)−(3) in order to choose regions in the selected countries, as the budget of the study did not allow for whole‐country coverage. The selection of regions resulted in Aquitaine (France), Lombardy (Italy) and Aragon (Spain) for maize, the Czech Republic (all regions), Eastern Germany and the East of England (UK) for OSR, and Andalusia (Spain) and the Northern Great Plain (Hungary) for sunflower. Hence, the results should be interpreted as being valid for these regions and not others.

**Table 1:** Main producers of target crops, CITF authorisations and derogations

Country	Production (million t)	Area (million ha)	CITF authorised products (pre‐restriction)	CITF derogations (post‐restriction)
*Maize*				
France	15.6	1.7	CT	‐
Italy	8.2	1.0	CIT	‐
Romania	6.0	2.7	C	CIT (2014‐16)
Germany	4.7	0.5	‐	‐
Hungary	4.2	1.2	CITF	CIT (2016)
Spain	4.2	0.4	CITF	‐
*OSR*				
France	5.4	1.6	‐	‐
Germany	4.8	1.3	CIT	‐
UK	2.6	0.8	CIT	CT (2015)
Poland	1.9	0.7	CI	‐
Czech Republic	1.1	0.4	CT	‐
Lithuania	0.6	0.3	CT	‐
*Sunflower*				
France	1.6	0.7	‐	‐
Romania	1.4	1.1	C	CIT (2014‐16)
Bulgaria	1.4	0.8	F	TF (2015), IT (2016)
Hungary	1.3	0.6	ITF	CIT (2016)
Spain	0.6	0.8	F	‐
Slovakia	0.2	0.1	CTF	‐

The six EU Member States with the largest production volume before the restrictions of each crop are listed. C, clothianidin; I, imidacloprid; T, thiamethoxam; F, fipronil. All derogations are for seed treatments. For maize and sunflower in Hungary, derogations are for seed production only. In the UK, 5% of the OSR area was derogated in 2015.

Sources: production and area are from the Food and Agriculture Organization of the United Nations (FAO; data for 2012).^55^ Information on authorised products is taken from the EFSA peer reviews of risk assessments for the active substances.^3^, ^4^, ^5^, ^7^ Information on derogations is from DG SANTE (personal communication).

In each of the selected eight regions, a farm survey was conducted. The target population of the surveys included all farmers who grew the selected crop and used at least one product containing CITF in one or more growing seasons before their use was restricted. The target population excluded farmers who had not used products containing CITF because those farmers were not directly affected by the restrictions, and in order to increase the statistical power for the farmers who did use CITF and were therefore directly affected. In the absence of census data on this target population, municipalities with a large presence of the target crop were identified with the assistance of regional and local agricultural authorities. At municipal offices, a list of sampling points, consisting of companies and organizations such as input dealers, crop buyers and distributors, and cooperatives, was created. Farmers were randomly contacted for interviews at these sampling points, and interviewed farmers sometimes provided the contact details of other farmers growing the target crop for additional interviews. Farmers who turned out to fall outside the target population were screened out.

The final dataset contains data from 100 farmers per region (800 in total) from the target population. Differences in certain characteristics between farmers in the samples and farmers in the target populations may create bias. One characteristic for which significant differences were found between the samples and official agricultural statistics was farm size. Farm size may also be correlated with differences in pest management practices. In order to improve representativeness, the distribution of farm size (hectares of arable land) in each survey was matched with the distribution of farm size in the selected region based on official national statistics. This matching was achieved in part by stratifying the sample before the survey, and, in addition, by weighting the data afterwards during data analysis.^47^, ^48^ For other farm characteristics, the absence of official statistical information on characteristics of the target population made further matching difficult. For example, detailed data on insecticide use including seed treatments are only available for the UK in its Pesticide Usage Survey (PUS) data, and even in this case, the published data are not integrated with other farm characteristics (such as farm size).^56^ The resulting datasets are thus representative of the farmers in each of the eight selected regions who grew the target crop and used a restricted substance before the restrictions, according to national farm size distributions. While correcting for farm size improves representativeness, we cannot guarantee representativeness according to additional characteristics.

Taylor Nelson Sofres (TNS, Madrid, Spain), a professional market research firm with an expertise in farmer surveys, was contracted to conduct the survey. The interviewers were trained and instructed to collect the most accurate information possible. The interviews were carried out after the end of the first growing season affected by the restrictions from the sowing date (because of the primary relevance of CITF as seed treatments). A first round of surveys (maize in Aquitaine, Lombardy and Aragon, and sunflower in the Northern Great Plain) was conducted in the spring of 2015. A second round of surveys (OSR in Eastern Germany, the East of England and the Czech Republic, and sunflower in Andalusia) was conducted in the autumn of 2015. This scheduling of the surveys was necessary to capture all relevant growing seasons: 2013/2014 winter crops were sown before the restrictions took effect in December 2013, whereas for summer crops the last season before the restrictions was 2013. Summer crops are sown and harvested within one calendar year, while winter crops are sown in one and harvested in the following calendar year. In this paper, we denote a growing season by one calendar year to allow easier reading and decided to use the calendar year in which a crop was sown. Hence the growing season of a winter crop sown in 2013 and harvested in 2014 is referred to as 2013, etc. Denoted in this way, the first growing season affected by the restrictions was 2014 for all (winter and summer) crops.

The interviews were guided by standardised questionnaires, which were designed to record general characteristics of farmers and their farms, as well as detailed data on agronomic practices and pest management (the full questionnaires are provided as Supporting Information Appendices S1 and S2). While additional data were collected to better understand the context of crop production operations, the primary variables of interest relate to the use of insecticides because they are most likely to be affected by the CITF restrictions. In addition, data on other changes in pest management, farmers' perceptions about the various impacts of the restrictions and valuations of CITF products, yield and insecticide cost were collected. In order to enable a sound evaluation of the restrictions, each farmer was asked to provide relevant data for the two growing seasons before, and the first season after the restrictions took effect.

The analysis of the collected data was conducted using Microsoft Excel and Stata (StataCorp, College Station, TX, USA). For the analysis of insecticide use, we used two main indicators, the first one being the proportion of farmers using a particular active substance, separately for seed treatments and soil/foliar treatments. To quantify soil and foliar applications in a single number, we calculated a treatment frequency index (TFI). The TFI has been used in similar contexts to quantify the intensity of pesticide use and is defined as the total number of (in our case insecticide) active substances applied per hectare during a growing season.^57^, ^58^ For example, two applications of cypermethrin and one application of lambda‐cyhalothrin would translate into a TFI of 3. Similarly, a product containing two active substances translates into a TFI of 2. We applied nonparametric tests of equality of matched pairs (Stata command *signtest*) to check for statistically significant differences in insecticide use between years. We also tested the data using the nonparametric Wilcoxon matched‐pairs signed‐ranks test (*signrank*), which yielded almost the same results. These tests cannot account for weights, which is why we also tested the data using paired *t*‐tests accounting for weights, even though the data were not normally distributed in many cases. However, the results from the paired *t*‐tests were mostly the same as those of the nonparametric tests. In the paper, we report the results of the nonparametric test of equality of matched pairs and provide the results from the *t*‐tests as supporting information (Tables S1 − S4). Other adaptations farmers have made in response to the restrictions, as well as farmers' perceptions of the impact of the restrictions on the time, cost and amount of plant protection products required to protect the crop, pest pressure, and the relative effectiveness of seed treatments, were analysed using the proportion of farmers as the main indicator. We summarised farmers' perceptions of pest pressure in one common indicator, which is defined as increasing if one type (soil or foliar) of pest pressure is increasing while the other is increasing or similar, decreasing if one type of pest pressure is decreasing while the other is decreasing or similar, and similar if both types of pest pressure are similar or one is increasing and the other decreasing. Tables 2–6 contain the key results, which are presented in the following section. Additional data are contained in Supporting Information Tables S5 − S8 and referred to in the last section of the paper.

**Table 2:** Insecticide seed treatments (% of farmers)

Substance or class	2012	2013	2014	2012	2013	2014	2012	2013	2014
	*Maize*								
	Aquitaine			Aragon			Lombardy		
Neonicotinoids (r)	87^a^	86^a^	0^b^	92^a^	93^a^	2^b^	14^a^	16^a^	16^a^
Neonicotinoids (u)	2^a^	5^a^	90^b^	2^a^	0^a^	78^b^			
Untreated seeds	26^a^	30^ab^	37^b^	0^a^	0^a^	10^b^	84^a^	81^a^	82^a^
	*OSR*								
	Czech Republic			Eastern Germany			East of England		
Neonicotinoids (r)	100^a^	94^b^	12^c^	99^a^	81^b^	3^c^	98^a^	98^a^	3^b^
Methiocarb	0^a^	1^a^	8^b^						
Untreated seeds	1^a^	6^a^	87^b^	1^a^	19^b^	97^c^	2^a^	3^a^	97^b^
	*Sunflower*								
	Andalusia			Northern Great Plain					
Neonicotinoids (r)	11^a^	11^a^	2^b^	92^a^	14^b^	1^c^			
Fipronil (r)	89^a^	85^a^	8^b^						
Tefluthrin	11^a^	16^a^	75^b^	1^a^	1^a^	3^a^			
Untreated seeds	18^a^	19^ab^	24^b^	7^a^	83^b^	88^b^			

The table shows the annual percentage of farmers by region who used untreated seeds or seeds treated with an insecticide product containing the mentioned active substance or an active substance belonging to the mentioned insecticide class. ‘(r)’ refers to the restricted substance clothianidin, imidacloprid, thiamethoxam or fipronil. ‘(u)’ refers to the unrestricted substance thiacloprid. Beta‐cyfluthrin is a common co‐formulant of clothianidin‐based seed treatment products, not used in isolation and therefore not separately shown. Columns may not sum to 100 if some farmers used more than one type of untreated or treated seeds, or some products were not known. Different superscript letters denote significantly different percentages of farmers between years within a region at the 5% level (test of equality of matched pairs). The data are from Q12 of the questionnaire.

## 3 RESULTS

### 3.1 Maize

#### 3.1.1 Aquitaine

Seeds treated with thiamethoxam were used by  > 85% of surveyed farmers in Aquitaine before the restrictions (Table 2). In 2012 and 2013, 11% and 9% of farmers used untreated seeds exclusively, while 15% and 20% used untreated seeds on some part (around 10%) of their maize area and neonicotinoid‐treated seeds on the remaining maize area. The use of seeds treated with thiacloprid, another neonicotinoid insecticide not affected by the restrictions, was marginal (2‐5%) in 2012 and 2013, but soared to 90% in 2014 when farmers stopped using thiamethoxam. The percentage of farmers using untreated seeds increased from 26‐30% to 37%.

Regarding the use of soil and foliar treatments, the most significant change was observed in pyrethroid use, which jumped from 22‐24% of farmers in 2012 and 2013 to 54% of farmers after the restrictions took effect in 2014 (Table 3). The increase in pyrethroid use was mainly for cypermethrin (from 15‐17% before to 49% after the restrictions). The most common cypermethrin‐based product was a granular formulation applied as a soil treatment during sowing. Farmers used it primarily for protection against wireworms (*Agriotes* spp.). Thiacloprid seed treatments also protect against wireworms, but they appear less effective than thiamethoxam‐treated seeds, which explains the additional use of cypermethrin‐based soil treatments.^59^ The farmers who had switched to thiacloprid were asked to compare the relative effectivenesses of the products. Sixty‐one per cent found the thiamethoxam‐based seed treatment more effective, while 36% regarded the treatments as similarly effective (Table 6).

**Table 3:** Insecticide soil and foliar treatments (% of farmers)

Substance or class	Soil/ foliar	2012	2013	2014	2012	2013	2014	2012	2013	2014
		*Maize*								
		Aquitaine			Aragon			Lombardy		
Neonicotinoids (r)	S							72^a^	28^b^	24^c^
Pyrethroids	S, F	22^a^	24^a^	54^b^				30^a^	54^b^	59^c^
Carbamates	S	24^a^	19^ab^	14^b^						
Chlorpyrifos	S, F				2^a^	14^ab^	19^b^	4^a^	3^a^	3^a^
Chlorantraniliprole	F, S	5^a^	10^a^	12^a^				1^a^	0^a^	0^a^
Other substances	F, S	7^a^	5^a^	5^a^				3^a^	3^a^	4^a^
No treatment		51^a^	47^b^	36^b^	97^a^	84^ab^	78^b^	16^a^	15^a^	15^a^
		*OSR*								
		Czech Republic			Eastern Germany			East of England		
Neonicotinoids (u)	F	47^a^	56^b^	62^b^	44^a^	45^a^	35^a^	0^a^	0^a^	8^b^
Pyrethroids	F, S	68^a^	81^b^	95^c^	60^a^	64^b^	86^c^	62^a^	59^a^	95^b^
Chlorpyrifos	F	52^a^	64^b^	74^c^						
Pymetrozin	F	2^a^	2^a^	3^a^	10^a^	3^a^	5^a^	0^a^	0^a^	3^a^
Indoxacarb	F	1^a^	1^a^	2^a^	4^a^	7^a^	6^a^			
No treatment					2^a^	1^a^	1^a^	38^a^	41^a^	5^b^
		*Sunflower*								
		Andalusia			Northern Great Plain					
Neonicotinoids (u)	F				1^a^	15^b^	19^b^			
Pyrethroids	F, S	5^a^	9^a^	11^a^	7^a^	39^b^	39^b^			
Carbamates	F				2^a^	11^b^	6^ab^			
Chlorpyrifos		10^a^	1^ab^	0^b^	2^a^	2^a^	2^a^			
Buprofezin					1^a^	0^a^	3^a^			
No treatment		87^a^	91^a^	89^a^	81^a^	32^b^	23^c^			

The table shows the annual percentage of farmers by region who used at least one insecticide product for soil or foliar application containing the mentioned active substance or an active substance belonging to the mentioned insecticide class. If S appears before F, the substance is more often used in soil than in foliar treatments, and vice versa. ‘(r)’ refers to the restricted substance clothianidin, imidacloprid or thiamethoxam. ‘(u)’ refers to the unrestricted substance acetamiprid or thiacloprid. Pyrethroids include alfa‐cypermethrin, beta‐cyfluthrin, esfenvalerate, etofenprox, gamma‐cyhalothrin, lambda‐cyhalothrin, cypermethrin, deltamethrin, tau‐fluvalinate, tefluthrin, zeta‐cypermethrin and a few other, less frequently mentioned active substances. Carbamates include methiocarb and pirimicarb. Other substances include diflubenzuron and abamectin. Columns may not sum to 100 if some farmers used more than one product, some products contained more than one active substance, or some products were not known. Different superscript letters denote significantly different percentages of farmers between years within a region at the 5% level (test of equality of matched pairs). The data are from Q26, Q34 and Q40 of the questionnaire.

Aquitaine maize farmers also used several other insecticides in soil and foliar treatments, but their use was not affected by the restrictions (Table 3). The proportion of farmers not using any soil or foliar treatment decreased after the restrictions from 51% to 36%. The average number of insecticide uses (TFI) increased significantly from 0.61 (2012) and 0.65 (2013) to 0.88 in 2014 (Table 4).

**Table 4:** Insecticide treatment frequency index (TFI)

	2012	2013	2014
Maize (Aquitaine)	0.61^a^	0.65^b^	0.88^c^
Maize (Aragon)	0.03^a^	0.16^ab^	0.24^b^
Maize (Lombardy)	1.18^a^	1.12^a^	1.10^a^
OSR (Czech Republic)	3.60^a^	3.86^b^	4.36^c^
OSR (Eastern Germany)	2.32^a^	2.40^a^	3.11^b^
OSR (East of England)	0.74^a^	0.72^a^	3.42^b^
Sunflower (Andalusia)	0.15^a^	0.10^ab^	0.11^b^
Sunflower (Northern Great Plain)	0.23^a^	0.74^b^	0.85^b^

The treatment frequency index (TFI) is the number of times each active substance is used, summed over all active substances. Different superscript letters indicate that values are significantly different at the 5% level (test of equality of matched pairs). The data are from Q26, Q34 and Q40 of the questionnaire.

Apart from insecticide use, 75% of farmers did not change any other aspect of pest management in response to the restrictions, although 22% of farmers scouted more frequently for pests (Table 5).

**Table 5:** Other adaptations (% of farmers)

	*Maize*			*OSR*			*Sunflower*	
	Aquitaine	Aragon	Lombardy	Czech Republic	Eastern Germany	East of England	Andalusia	Northern Great Plain
Increased sowing density	5	6	3	8	45	12		26
Earlier sowing date	3	2	20	3	2	61	2	5
Later sowing date	5		1		47	1		8
Reduced crop area		1	1	1	5	1	3	9
More mechanical control		6	7		2	30	1	20
More pest scouting	22	10	9	54	64	25	16	12
None	75	77	63	39	21	26	88	43

The table shows the percentage of farmers in each region who used a particular adaptation measure in response to the restrictions. Columns for some countries may not sum to 100 because some farmers used more than one adaptation measure. The data are from Q47 of the questionnaire.

Regarding their perceptions of the impact of the restrictions on pest management, 37% of farmers reported larger time requirements for crop protection, which can be explained by the increased use of insecticides and more frequent pest scouting (Table 6). The rest did not perceive any effect on required time, which is probably because their most common adaptation to the restrictions involved a switch from one seed treatment to another. Regarding pest management costs, 57% of farmers perceived an increase, which can be explained by higher insecticide use.

**Table 6:** Farmer perceptions of impact of restrictions (% of farmers)

	*Maize*			*OSR*			*Sunflower*	
	Aquitaine	Aragon	Lombardy	Czech Republic	Eastern Germany	East of England	Andalusia	Northern Great Plain
Crop protection requires: more ‐ similar ‐ less								
Time	37 ‐ 63 ‐ 0	40 ‐ 60 ‐ 0	13 ‐ 87 – 0	76 ‐ 21 ‐ 0	93 ‐ 7 ‐ 0	81 ‐ 19 ‐ 0	19 ‐ 81 ‐ 0	74 ‐ 25 ‐ 0
Cost	57 ‐ 37 ‐ 6	59 ‐ 33 ‐ 8	11 ‐ 89 – 0	79 ‐ 16 ‐ 0	83 ‐ 14 ‐ 0	84 ‐ 14 ‐ 2	32 ‐ 68 ‐ 0	73 ‐ 23 ‐ 4
Insecticides	44 ‐ 54 ‐ 2	43 ‐ 56 ‐ 0	10 ‐ 90 – 0	77 ‐ 22 ‐ 0	85 ‐ 11 ‐ 0	81 ‐ 19 ‐ 0	11 ‐ 89 ‐ 0	70 ‐ 28 ‐ 2
Effectiveness of restricted vs replacement seed treatment:								
Higher ‐ equal ‐ lower	61 ‐ 36 ‐ 3	79 ‐ 20 ‐ 0	‐	‐	‐	‐	44 ‐ 50 ‐ 5	‐
Pest pressure:								
Higher ‐ similar ‐ lower	40 ‐ 49 ‐ 11	80 ‐ 14 ‐ 6	5 ‐ 95 – 0	68 ‐ 22 ‐ 10	77 ‐ 23 ‐ 0	68 ‐ 32 ‐ 0	1 ‐ 99 ‐ 0	51 ‐ 46 ‐ 3

The data are from Q48 − 52 of the questionnaire.

Perceptions of pest pressure before and after the restrictions were similar for about half of the farmers (Table 6). However, 40% perceived an increase, while 11% perceived a decrease. Pests with higher perceived pressure include mostly wireworms and corn borers (*Ostrinia nubilalis* and *Sesamia nonagrioides*).

#### 3.1.2 Aragon

Before the restrictions, >90% of the surveyed Aragon farmers used seeds treated with clothianidin and 6% used seeds treated with thiamethoxam (Table 2). A small number of farmers used treated seeds but either did not remember the product or active substance, or preferred not to name it. None of the surveyed farmers used untreated seeds. After the restrictions in 2014, all but 2% of farmers had stopped using seeds treated with clothianidin or thiamethoxam. Similar to the Aquitaine case, most Aragon farmers (78%) switched to using thiacloprid‐treated seeds, and also most of the Aragon farmers who made this switch rated thiacloprid as less effective than clothianidin or thiamethoxam (79%), while 20% perceived no difference (Table 6). The proportion of farmers using untreated seeds rose from 0% to 10%.

Regarding soil and foliar insecticides, almost no maize farmers reported having used them prior to 2014, in contrast with Aquitaine (and Lombardy) farmers (Table 3). One reason could be that the uptake of corn borer‐resistant *Bacillus thuringiensis* (*Bt*) maize varieties is very high in the region, which can explain a lower use of other insecticides.^60^, ^61^ We observed an increase in the proportion of maize farmers using the organophosphate chlorpyrifos (applied as a soil treatment during sowing) already before 2014 (2% in 2012 to 14% in 2013 to 19% in 2014), along with a decrease in the number of farmers not applying any soil/foliar treatments (97% in 2012 to 84% in 2013 to 78% in 2014). The TFI also increased from 0.03 in 2012 to 0.16 in 2013 to 0.24 in 2014 (Table 4). The pests targeted with chlorpyrifos commonly include wireworms, so there is an overlap with the target pests of neonicotinoid seed treatments. But as the trend of increasing insecticide usage was occurring already between 2012 and 2013, it is not clear if it is linked to the restrictions.

As in Aquitaine, the majority (77%) of Aragon maize farmers did not make any other changes to their pest management (Table 5). More pest scouting was done by 10% of farmers; several other adaptations were mentioned by only very few farmers.

The perceptions of Aragon farmers of the impact of the restrictions on crop protection requirements were similar to those of Aquitaine farmers (Table 6). Forty per cent said crop protection required more time, while 60% saw no difference as most farmers simply switched to seeds treated with a different insecticide. About 60% of farmers, however, saw an increase in cost, which is not easily explained because reported cost for treated seeds was similar for clothianidin, thiamethoxam and thiacloprid and farmers did not change any other aspects of pest management. The overall amount of chemical plant protection products required for crop protection was perceived to be increasing as a result of the restrictions for 43% of farmers. Again, it is not clear why a significant number of farmers perceived an increase when the reported insecticide use did not show a clear parallel trend. Pest pressure increased after the restrictions according to 80% of farmers (Table 6). This can be explained by the lower effectiveness of thiacloprid compared with clothianidin and thiamethoxam.^59^ Major pests with increased perceived pressure include wireworms, cutworms (*Agrotis* spp.) and leafworms (especially *Spodoptera littoralis*).

#### 3.1.3 Lombardy

Lombardy is a special case in our study, as the use of seed treatments with neonicotinoids had already been prohibited there since 2008, several years before the EU restrictions (however, soil and foliar treatments with neonicotinoids were not prohibited). As expected, we found that the large majority (> 80%) of farmers were already using untreated seeds before the EU restrictions, and this was not further affected in 2014 (Table 2). However, about 15% of surveyed farmers were using seeds treated with neonicotinoids (clothianidin, imidacloprid, or thiamethoxam) before and after the EU restrictions, and it is unclear why farmers were able to do this. Evidence for the use of restricted neonicotinoid seed treatments in Italy was also provided by Sabatino *et al*. (2013).^11^ One potential explanation is that Italian farmers are not strongly integrated into cooperatives, making control and enforcement of restrictions more difficult.

Most of the surveyed Lombardy farmers used soil and foliar treatments (Table 3). In 2012, >70% of farmers were using a clothianidin‐based soil treatment when drilling maize. Corn borers, cutworms and wireworms were typically named as target pests of the clothianidin‐based soil treatments. Thirty per cent of farmers used a pyrethroid soil treatment in 2012, in most cases either deltamethrin or tefluthrin, targeting these same pests. However, this proportion of farmers increased to 54% in 2013, while the proportion using the neonicotinoid‐based soil treatment decreased to 28%. According to local plant protection authorities contacted during the survey, this can be explained because farmers were encouraged to reduce the use of neonicotinoid soil treatments already before the EU restrictions. However, neonicotinoid soil treatments did not disappear and their use continued at this lower proportion of farmers in 2014, after the EU restrictions, similar to observations for seed treatments. No other large changes in insecticide use were observed in Lombardy. The TFI was between 1.1 and 1.2, with no statistically significant difference between the years (Table 4).

Over 60% of Lombardy farmers surveyed did not take additional pest management measures in response to the EU restrictions (Table 5), while 20% of farmers switched to an earlier sowing date. Several other adaptations, including more mechanical pest control and more pest scouting, were mentioned by <10% of farmers. Ninety‐five per cent of farmers did not perceive any changes in pest pressure after the restrictions (Table 6). Nearly 90% of farmers did not perceive any effect of the CITF restrictions on the time, cost, and amount of chemical plant protection products required to protect their crop.

### 3.2 Oilseed rape

#### 3.2.1 Czech Republic

Seeds treated with neonicotinoids were used by nearly all Czech farmers in the survey before the restrictions (Table 2). Most farmers (85‐86%) used thiamethoxam‐treated seeds, while imidacloprid‐ and clothianidin‐based seed treatments (with beta‐cyfluthrin as a common co‐ingredient) were used to a smaller extent. After the restrictions, the use of neonicotinoid‐based seed treatments was reduced to 12% of farmers, while 87% of farmers were using untreated seeds, up from 6% in 2013. The use of methiocarb‐treated seeds slightly increased from 1% to 8%. It is not clear why 12% of farmers were still using restricted neonicotinoids in 2014, but one possibility is the use of old seed stocks.

The use of foliar insecticides was common for all surveyed farmers in all years (Table 3). Neonicotinoids, pyrethroids and organophosphates were widely used before and after the restrictions. Active substances commonly applied include acetamiprid and thiacloprid (neonicotinoids not affected by the restrictions), chlorpyrifos (organophosphate), and cypermethrin, deltamethrin, gamma‐cyhalothrin and several other pyrethroids. In many cases, the products used contain two active substances, for example chlorpyrifos plus cypermethrin or deltamethrin plus thiacloprid. Farmers targeted a large number of pests including most commonly the cabbage stem flea beetle (CSFB; *Psylliodes chrysocephala*), the cabbage stem weevil (*Ceutorhynchus pallidactylus*) and the pollen beetle (*Meligethes aeneus*), and, to a lesser extent, the cabbage root fly (*Delia radicum*), the cabbage seed weevil (*Ceutorhynchus assimilis*), the rape stem weevil (*Ceutorhynchus napi*) and the brassica pod midge (*Dasineura brassicae*). The proportion of farmers using foliar treatments increased between 2012 and 2014, with neonicotinoid use increasing from <50% to >60% of farmers, pyrethroid use from <70% to >90%, and chlorpyrifos use from 52% to 74%. However, this significant increase in foliar treatments started in 2013, a year before the restrictions were enacted. The increase in insecticide use intensity is also reflected in the TFI (Table 4), which showed an increase from 3.6 in 2012 to 3.9 in 2013 to 4.4 in 2014. It is therefore difficult to estimate if all or only part of the total increase was attributable to the neonicotinoid restrictions.

Taking into account the perceptions of Czech farmers (Table 6), 77% stated that a higher amount of plant protection products was required because of the restrictions. Pest pressure was reported to have increased after the restrictions by almost 70% of farmers (primarily from CSFB). More than 50% of the farmers did more pest scouting in response to the restrictions, while other adaptation measures were not prominent (Table 5). Close to 80% of farmers also perceived that the restrictions made protecting their crop more time‐ and cost‐intensive.

#### 3.2.2 Eastern Germany

Neonicotinoid‐treated seeds were widely used by farmers in Eastern Germany before the restrictions took effect, and practically all of them switched to using untreated seeds afterwards. The main seed treatment used by >90% of farmers contained the active substances clothianidin and beta‐cyfluthrin (Table 2). Imidacloprid and thiamethoxam were used to a much smaller extent. The restrictions might have had an impact on neonicotinoid seed treatment usage already in 2013 (81% compared with 99% in 2012). It is possible that some seed companies limited the production of treated seeds in 2013 to reduce the risk of being left with stocks that might no longer be marketable in case the restrictions took effect early.

Foliar insecticide treatments were widely used before the restrictions took effect, including a number of different pyrethroids (most prominently lambda‐cyhalothrin, alfa‐cypermethrin, beta‐cyfluthrin and etofenprox, among others) and the unrestricted neonicotinoids thiacloprid and (to a smaller extent) acetamiprid (Table 3). Products containing the organophosphate chlorpyrifos, while widely used by OSR farmers in the Czech Republic, were not authorised for OSR in Germany. The most frequent target pests include CSFB, the pollen beetle, the rape stem weevil and the cabbage root fly. The use of neonicotinoid foliar treatments did not change significantly with the restrictions; however, the proportion of farmers using foliar treatments with pyrethroids increased significantly from 64% before to 86% after the restrictions. A proportion of farmers also reported the use of insecticides which they did not specify (the reason is not clear). Such unknown insecticides were used by 15‐18% of farmers before and 27% of farmers after the restrictions, and often exclusively (no known insecticides were used in the same season), which means that the increase in the proportion of farmers using a particular substance or insecticide class (such as pyrethroids) was even higher than that shown in Table 3.

The TFI was 2.3 − 2.4 before and 3.1 after the restrictions came into effect (Table 4). In 2014, the proportion of farmers spraying insecticides only once fell from >30% to 12%, while the most common number of applied insecticides was 3 (31% of farmers). The median number of insecticides applied was 2 in 2012 and 2013, and 3 in 2014. CSFB was targeted with sprays by about 80% of farmers in 2014, while only 23% sprayed against it in 2012 and 35% in 2013. About 33% of farmers sprayed to control cabbage root fly in 2014, whereas only 3‐5% of farmers did in 2012 and 2013.

Around 80% of farmers also modified other pest management practices in response to the restrictions (Table 5). Over 60% scouted more often for pests, 47% delayed the sowing date and 45% increased the sowing density. All three measures were also recommended to farmers as a response to the restrictions.^62^ For a large majority of farmers (>80%), the restrictions meant increasing time, cost and insecticide requirements for crop protection (Table 6). Seventy‐seven per cent of farmers perceived a higher pest pressure, most commonly including CSFB and cabbage root fly.

Our results are in line with the observation of increased insecticide use by Market Probe (2015a, 2015b).^40^, ^41^ Production cost increases and more frequent pest scouting were also found by Market Probe (2015a, 2015b) for a majority of farmers. According to Market Probe (2015a, 2015b), between 0% and 29% (depending on question phrasing) of farmers reduced the area of OSR grown as a consequence of the restrictions, and our result of 5% is inside, albeit towards the lower end, of that range (Table 5).

#### 3.2.3 East of England

Similarly to the Czech Republic and Eastern Germany, almost all farmers surveyed in the East of England used neonicotinoid‐treated seeds before the restrictions (Table 2). Around 80% of farmers used seeds treated with thiamethoxam, while clothianidin‐/beta‐cyfluthrin‐ and imidacloprid‐treated seeds were less common. This distribution is broadly in line with the data on the last season before the restrictions from the PUS statistics, although thiamethoxam use may be somewhat overrepresented.^56^ After the restrictions, the use of treated seeds almost disappeared, while the proportion of farmers using untreated seeds increased from just 2‐3% in 2012 and 2013 to 97% in 2014.

The area‐corrected share of thiamethoxam among all treated hectares in our survey of the East of England was 81% before the restrictions and thus higher than the 58% reported for the so‐called eastern region in the PUS data. Hence, there may be a concern that a potential overrepresentation of thiamethoxam users may introduce bias in our results. As the PUS data are not available by farm size, which would allow weighting the data accordingly, we instead checked, for all outcome variables, whether former users of thiamethoxam behaved differently after the restrictions from former users of clothianidin. We found a significant difference for area reduction (highlighted below), but not for any other outcome variable. This suggests that former users of thiamethoxam‐ and clothiandin‐treated seeds behaved broadly similarly in response to the restrictions.

Regarding foliar treatments, around 60% of farmers used pyrethroids before the restrictions, while in 2014 this proportion increased to 95% (Table 3). Two active substances were mainly used: cypermethrin (its use rose from 30% of farmers to >80% after the restrictions) and lambda‐cyhalothrin (its use rose from 33‐35% to almost 70% of farmers after the restrictions). The target pests include, most commonly, CSFB, and, to a smaller extent, the pollen beetle and the peach potato aphid (*Myzus persicae*), among others. Products containing the organophosphate chlorpyrifos, while widely used by OSR farmers in the Czech Republic, were not authorised for OSR in the UK.

The TFI increased almost five‐fold from around 0.7 to 3.4 (Table 4). The median number of insecticides applied increased from 1 in 2013 to 3 in 2014. Eighty‐seven per cent of farmers were applying one or no insecticides in 2013. In 2014, 89% of farmers were applying two or more insecticides. Before the restrictions, around 40% of farmers were not applying any foliar treatments, and this proportion fell to 5% afterwards.

Additional changes in pest management were made by 74% of farmers (Table 5). The most common change for 61% of farmers was an earlier sowing date. According to Wynn *et al*. (2014), an earlier sowing date was widely perceived as reducing the susceptibility to CSFB. Earlier sowing dates in the East of England are in contrast with the later sowing dates we found in eastern Germany. In Germany, later sowing dates were recommended to reduce the susceptibility to the cabbage root fly.^62^ Thirty per cent of farmers also mentioned mechanical pest control, and 25% more frequent pest scouting. What concrete measures are meant by mechanical pest control was not further specified, but our result may be related to the finding of Wynn *et al*. (2014) of farmers responding to the restrictions with the preparation of better seed beds.^39^ Sowing density was increased by 12% of farmers. Over 80% of farmers perceived the restrictions as increasing the time, cost and amount of insecticides required to protect their crop (Table 6). Pest pressure was perceived to increase for 68% of farmers, while it remained similar for 32%. The most commonly mentioned pest with an increasing incidence was CSFB.

Unlike most other case studies presented in this paper, for which no previous analyses of the impact of restrictions on pest management are available, the case of OSR in the UK has been the subject of studies based on expert opinion and farm surveys. Before comparing our results to other published data, it is worth noting that our survey covered most of the counties in the East of England that Wynn *et al*. (2014) identified as the most affected by pest damage in September 2014.^39^

Our data largely confirm the findings of increased foliar insecticide applications and increased pest pressure.^39^, ^44^, ^45^, ^47^, ^50^, ^51^, ^53^, ^54^ One generally very useful source of information on insecticide use in the UK is the PUS data.^56^ However, the PUS are not conducted on an annual basis. PUS data are available for the last season before the restrictions.^63^ Data covering the first season after the restrictions were not collected. Data covering the second seasons after the restrictions have been collected but were not yet published at the time of writing this manuscript.

Changes in planting dates and pest monitoring were also reported by Wynn *et al*. (2014).^39^ Increased time and cost requirements for crop protection are in line with the results of Market Probe (2015e, 2015f) and Zhang *et al*. (2017).^44^, ^45^, ^54^ Some of the cited studies suggest that 5‐19% (depending on how the question was asked) of farmers reduced their OSR area as a result of the restrictions, while in our survey <5% of farmers reduced OSR area in response to the restrictions (even when correcting for the overrepresentation of former thiamethoxam users).^44^, ^45^, ^46^

### 3.3 Sunflower

#### 3.3.1 Andalusia

Seeds treated with fipronil were used by most farmers before the restrictions (89% in 2012 and 85% in 2013), while 11% of farmers used seeds treated with thiamethoxam (Table 2). After the restrictions, the majority of farmers switched to seeds treated with the pyrethroid tefluthrin, with 75% of farmers using such seeds in 2014 compared with 11‐16% before, while fipronil use declined to 8% (perhaps using up old stock). Around 20% of farmers used untreated seeds but that proportion changed little with the restrictions.

The large majority (about 90%) of farmers did not use additional soil or foliar treatments, and this was not significantly affected by the restrictions (Table 3). The few farmers using insecticides reported the pyrethroid deltamethrin and the organophosphate chlorpyrifos, with cutworms and wireworms being the main relevant target pests. These pests are also targeted by fipronil and pyrethroid seed treatments. The TFI remained low at 0.1‐0.15, with no significant change caused by the restrictions (Table 4). A large majority of 88% of farmers declared no other adaptations in pest management in response to the restrictions (Table 5).

Consistent with the above, 80‐90% of farmers perceived no impact on the time or amount of insecticides required to protect their crop (Table 6). Most farmers (68%) did not perceive an impact on cost either, but 32% saw an increase in cost, the reason not being entirely clear. Furthermore, roughly half of the farmers who switched from a restricted to an unrestricted seed treatment regarded the tefluthrin seed treatments as less effective than the restricted fipronil and neonicotinoid seed treatments, with the other half noting no difference. Still, 99% of farmers perceived a similar incidence of pests before and after the restrictions.

#### 3.3.2 Northern Great Plain

More than 90% of Northern Great Plain farmers used neonicotinoid‐treated seeds in 2012, and by 2014, this proportion decreased to 1% (Table 2). Untreated seeds were not commonly used in 2012, but almost 90% of farmers relied on them in 2014. Unlike the case of Spain, tefluthrin was not authorised for seed treatments in sunflower in Hungary and therefore not available to farmers. The neonicotinoid seed treatments used before the restrictions were thiamethoxam, used by 76% of farmers in 2012, and imidacloprid, used by 26% of farmers in 2012 (a few farmers used both insecticides on different parts of their area). The switch from neonicotinoid‐treated to untreated seeds occurred already in 2013. An explanation is that seed companies, anticipating the restrictions without necessarily knowing when they might come into effect, did not produce sufficient amounts of treated seed, in order to avoid being left with stocks that might no longer be marketable. Thus, the restrictions already had their major impact before they came into force. Consequently, farmers also began modifying the use of foliar treatments in 2013 (Table 3). The proportion of farmers applying foliar insecticides rose from 19% in 2012 to 68% in 2013 (and increased further to 77% in 2014). The main insecticides used were pyrethroids, used by 7% of farmers in 2012 and 39% in 2013 and 2014. The percentage of farmers using foliar applications of the unrestricted neonicotinoid thiacloprid also increased from 1% in 2012 to 15‐19% in 2013 and 2014. These treatments most commonly targeted pests including *Heliothis* spp., the weevil *Tanymecus dilaticollis*, the sunflower moth (*Homoeosoma nebulella*), cutworms, wireworms and white grubs (*Melolontha* spp.). The TFI more than tripled from 0.2 in 2012 to 0.7 in 2013 and 0.9 in 2014 (Table 4).

Other adaptations to the restrictions were made by 57% of farmers (Table 5). The most common measure was an increase in sowing density (26% of farmers), followed by more mechanical pest control (not further specified). Twelve per cent of farmers also engaged in more frequent pest scouting. Nine per cent declared to have reduced the area on which they grow sunflower in response to the restrictions. In addition, 4% of farmers did not grow sunflower in 2014 at all, citing the non‐availability of seed treatments as the main reason.

Over 70% of farmers perceived the restrictions as increasing time, cost and insecticide requirements for crop protection, while the rest perceived no difference (Table 6). This perception can be linked to the increased use of insecticides and other changes in pest management. Farmers were almost evenly divided between perceiving that the restrictions increased and did not affect pest pressure. Farmers who reported an increase in pest pressure most often mentioned white grubs and aphids.

## 4 DISCUSSION

The use of CITF seed treatments to manage soil‐dwelling and early‐season pests was commonplace in most of the surveyed crops and regions before the restrictions. The response to the restrictions by farmers has been less uniform, with significant differences between crops and regions.

In four case studies (OSR in the Czech Republic, Eastern Germany and the East of England, and sunflower in the Northern Great Plain), alternative insecticide seed treatments were not available, and farmers switched to using untreated seeds. To compensate for the loss of CITF seed treatments, farmers relied mostly on increasing foliar treatments. This increase also showed variability, being particularly pronounced in the East of England and the Northern Great Plain. Other changes in pest management were also frequent in this group of case studies, often including changes in sowing date, a higher sowing density and more pest scouting. Consequently, most farmers perceived that the time, cost and insecticide requirements of crop protection had increased.

The second group of case studies (maize in Aquitaine and Aragon and sunflower in Andalusia) was characterised by the availability of alternative insecticides for seed treatments. Farmers mostly switched to unrestricted neonicotinoid‐ or pyrethroid‐treated seeds. Therefore, changes in the use of other insecticides were less common (with the exception of pyrethroid soil treatments in Aquitaine). Most farmers did not change other pest management practices, and the perceived impact of the restrictions on time, cost and insecticide use was less pronounced than in the first group of case studies. Impacts of neonicotinoid restrictions have also been less significant in the special case of Lombardy, where neonicotinoid seed treatments had been restricted since 2008 and neonicotinoid soil treatments were already reduced and replaced by pyrethroid soil treatments before the EU restrictions.

This is the first study detailing changes in insecticide use in response to the CITF restrictions in maize and sunflower. For OSR, studies in the UK (and one in Germany) also reported a lack of alternative seed treatments and increases in foliar treatments, which we confirm based on our comprehensive dataset on insecticide use over three consecutive growing seasons.^40^, ^41^, ^44^, ^45^, ^47^, ^51^ An increased reliance on a more limited range of insecticides with the same mechanism of action, such as pyrethroids, may worsen issues associated with insecticide resistance.^64^ Our results also confirm previous studies of OSR in Germany and the UK which found changes in the sowing date, a higher sowing density and more pest scouting. Increased scouting may result in less insecticide use (particularly if pest levels are below the economic threshold). Reports of a reduction in the OSR grown in Germany and the UK in response to the CITF are only partially confirmed.^44^, ^45^, ^46^ Some studies from the UK also found that small parts of the OSR area were sown but the crop failed to establish and they were not successfully redrilled.^46^, ^47^ While we did not collect data on establishment failure directly, increased sowing densities reported by many farmers (particularly in Eastern Germany and the Northern Great Plain) indicate that farmers took measures to avoid it.

The effectiveness of alternatives to CITF in controlling pests has often been discussed. We made no attempt to directly measure the effectiveness of the alternatives found in our survey, but farmers tended to perceive alternative seed treatments as less effective. Another relevant result is the perception of changes in pest pressure. Generally, farmers perceived an increase in pest pressure after the restrictions and indicated the pests involved; only in two case studies pest pressure was perceived to be similar. A question on the perception of changes in the incidence of wild beneficial insects was also included and the large majority of farmers (90–100% in seven of eight case studies) did not perceive any changes (Table S5). However, this question is considered to have limited value as farmers in many cases may not be trained in making such assessments (as opposed to monitoring pests).

A much‐debated question concerns the impact of the CITF restrictions on productivity and economic outcomes for farmers. Several studies (primarily on OSR in the UK) looked at the potential impact of the restrictions on yield, with results ranging from negative to no effects, depending on the study and the region.^40^, ^41^, ^44^, ^45^, ^53^, ^54^ In our survey, we asked farmers for yield data for growing seasons before and after the CITF restrictions, which showed small and significant changes after the restrictions in some of the examined regions, but not in others (Table S6). However, no conclusions from this data on the specific effect of the CITF restrictions can be drawn because of the multi‐factorial nature of yields. In terms of cost, the general perception of farmers is that the restrictions have made crop protection more time‐ and cost‐intensive. This perception is quite variable but clearly higher in the case studies where no alternative seed treatments were available. In a few case studies we obtained enough data on the reported cost of insecticide applications per growing season. For example, in Aquitaine this cost significantly increased from €19 ha^‐1^ before to €29 ha^‐1^ after the restrictions (not shown), but in most cases the response rate to this question was too low to reliably estimate cost changes. Another approach to estimating the economic impact of the restrictions is to look at farmers' valuations of CITF products. Most farmers in each of the eight regions attached a positive monetary value to the restricted CITF products, and this valuation was higher in some regions than in others (Table S7). Farmers valued different characteristics of the restricted products, most often their effectiveness, the ease of using them, and the fact that they often make other insecticide treatments unnecessary (Table S8).

The results reported here are valid for farmers in the eight regions examined and might be different in other regions of the seven examined countries, as well as in other countries. Another aspect not addressed by our study is temporal pest population dynamics, which could have effects on other farmers and crops as well. Such dynamics, together with potential resistance development, learning by farmers and changing availability of insecticides mean that the pest management changes observed for the first year after the restrictions cannot necessarily be extrapolated to subsequent growing seasons, which require further study.^48^

## Supporting information

Supplementary Tables

Questionnaire Round 1

Questionnaire Round 2
