# Supplementary material for: The impact of restrictions on neonicotinoid and fipronil insecticides on pest management in maize, oilseed rape and sunflower in eight European Union regions
Source: Pest Manag Sci. 2017 Oct 13;74(1):88–99. doi: 10.1002/ps.4715 (PMC5765491; doi:10.1002/ps.4715)
Supplement: Supplementary file 1 — Supplementary Tables [file PS-74-88-s003.docx]

**SUPPORTING INFORMATION**

**Table S1: Insecticide seed treatments (% of farmers) – using t-tests**

| Substance or class | 2012 | 2013 | 2014 | 2012 | 2013 | 2014 | 2012 | 2013 | 2014 |
| --- | --- | --- | --- | --- | --- | --- | --- | --- | --- |
|  | *Maize* | | | | | | | | |
|  | Aquitaine | | | Aragon | | | Lombardy | | |
| Neonicotinoids (r) | 87^a^ | 86^a^ | 0^b^ | 92^a^ | 93^a^ | 2^b^ | 14^a^ | 16^a^ | 16^a^ |
| Neonicotinoids (u) | 2^a^ | 5^a^ | 90^b^ | 2^a^ | 0^a^ | 78^b^ |  |  |  |
| Untreated seeds | 26^a^ | 30^a^ | 37^a^ | 0^a^ | 0^a^ | 10^a^ | 84^a^ | 81^a^ | 82^a^ |
|  | *OSR* | | | | | | | | |
|  | Czech Republic | | | Eastern Germany | | | East of England | | |
| Neonicotinoids (r) | 100^a^ | 94^b^ | 12^c^ | 99^a^ | 81^b^ | 3^c^ | 98^a^ | 98^a^ | 3^b^ |
| Methiocarb | 0^a^ | 1^a^ | 8^b^ |  |  |  |  |  |  |
| Untreated seeds | 1^a^ | 6^a^ | 87^b^ | 1^a^ | 19^b^ | 97^c^ | 2^a^ | 3^a^ | 97^b^ |
|  | *Sunflower* | | | | | |  | | |
|  | Andalusia | | | Northern Great Plain | | |  |  |  |
| Neonicotinoids (r) | 11^a^ | 11^a^ | 2^b^ | 92^a^ | 14^b^ | 1^c^ |  |  |  |
| Fipronil (r) | 89^a^ | 85^a^ | 8^b^ |  |  |  |  |  |  |
| Tefluthrin | 11^a^ | 16^a^ | 75^b^ | 1^a^ | 1^a^ | 3^a^ |  |  |  |
| Untreated seeds | 18^a^ | 19^a^ | 24^a^ | 7^a^ | 83^b^ | 88^b^ |  | | |
| The table shows the annual percentage of farmers by region who used untreated seeds or seeds treated with an insecticide product containing the mentioned active substance or an active substance belonging to the mentioned insecticide class. ‘(r)’ refers to the restricted substance clothianidin, imidacloprid, thiamethoxam or fipronil. ‘(u)’ refers to the unrestricted substance thiacloprid. Beta-cyfluthrin is a common co-formulant of clothianidin-based seed treatment products, not used in isolation and therefore not separately shown. Columns may not sum to 100 if some farmers used more than one type of untreated or treated seeds, or some products were not known. Different superscript letters denote significantly different percentages of farmers between years within a region at the 5% level (paired t-test). The data are from Q12 of the questionnaire. | | | | | | | | | |

**Table S2: Insecticide soil and foliar treatments (% of farmers) –using t-tests**

| Substance or class | Soil/ foliar | 2012 | 2013 | 2014 | 2012 | 2013 | 2014 | | 2012 | 2013 | 2014 |
| --- | --- | --- | --- | --- | --- | --- | --- | --- | --- | --- | --- |
|  | | *Maize* | | | | | | | | | |
|  |  | Aquitaine | | | Aragon | | | | Lombardy | | |
| Neonicotinoids (r) | S |  |  |  |  |  |  | | 72^a^ | 28^b^ | 24^c^ |
| Pyrethroids | S, F | 22^a^ | 24^a^ | 54^b^ |  |  |  | | 30^a^ | 54^b^ | 59^b^ |
| Carbamates | S | 24^a^ | 19^ab^ | 14^b^ |  |  |  | |  |  |  |
| Chlorpyrifos | S, F |  |  |  | 2^a^ | 14^ab^ | | 19^b^ | 4^a^ | 3^a^ | 3^a^ |
| Chlorantraniliprole | F, S | 5^a^ | 10^a^ | 12^a^ |  |  | |  | 1^a^ | 0^a^ | 0^a^ |
| Other substances | F, S | 7^a^ | 5^a^ | 5^a^ |  |  | |  | 3^a^ | 3^a^ | 4^a^ |
| No treatment | | 51^a^ | 47^ab^ | 36^b^ | 97^a^ | 84^ab^ | | 78^b^ | 16^a^ | 15^a^ | 15^a^ |
|  | | *OSR* | | | | | | | | | |
|  |  | Czech Republic | | | Eastern Germany | | | | East of England | | |
| Neonicotinoids (u) | F | 47^a^ | 56^b^ | 62^b^ | 44^a^ | 45^a^ | 35^a^ | | 0^a^ | 0^a^ | 8^b^ |
| Pyrethroids | F, S | 68^a^ | 81^b^ | 95^c^ | 60^a^ | 64^b^ | 86^c^ | | 62^a^ | 59^a^ | 95^b^ |
| Chlorpyrifos | F | 52^a^ | 64^b^ | 74^c^ |  |  |  | |  |  |  |
| Pymetrozin | F | 2^a^ | 2^a^ | 3^a^ | 10^a^ | 3^a^ | | 5^a^ | 0^a^ | 0^a^ | 3^a^ |
| Indoxacarb | F | 1^a^ | 1^a^ | 2^a^ | 4^a^ | 7^a^ | | 6^a^ |  |  |  |
| No treatment | |  |  |  | 2^a^ | 1^a^ | | 1^a^ | 38^a^ | 41^a^ | 5^b^ |
|  | | *Sunflower* | | | | | | |  | | |
|  |  | Andalusia | | | Northern Great Plain | | | |  |  |  |
| Neonicotinoids (u) | F |  |  |  | 1^a^ | 15^b^ | 19^b^ | |  |  |  |
| Pyrethroids | F, S | 5^a^ | 9^a^ | 11^a^ | 7^a^ | 39^b^ | 39^b^ | |  |  |  |
| Carbamates | F |  |  |  | 2^a^ | 11^b^ | 6^ab^ | |  |  |  |
| Chlorpyrifos |  | 10^a^ | 1^ab^ | 0^b^ | 2^a^ | 2^a^ | 2^a^ | |  |  |  |
| Buprofezin |  |  |  |  | 1^a^ | 0^a^ | | 3^a^ |  |  |  |
| No treatment | | 87^a^ | 91^a^ | 89^a^ | 81^a^ | 32^b^ | | 23^c^ |  |  |  |
| The table shows the annual percentage of farmers by region who used at least one insecticide product for soil or foliar application containing the mentioned active substance or an active substance belonging to the mentioned insecticide class. If S appears before F, the substance is more often used in soil than in foliar treatments, and vice versa. ‘(r)’ refers to the restricted substance clothianidin, imidacloprid or thiamethoxam. ‘(u)’ refers to the unrestricted substance acetamiprid or thiacloprid. Pyrethroids include alfa-cypermethrin, beta-cyfluthrin, esfenvalerate, etofenprox, gamma-cyhalothrin, lambda-cyhalothrin, cypermethrin, deltamethrin, tau-fluvalinate, tefluthrin, zeta-cypermethrin and a few other, less frequently mentioned active substances. Carbamates include methiocarb and pirimicarb. Other substances include diflubenzuron and abamectin. Columns may not sum to 100 if some farmers used more than one product, some products contained more than one active substance, or some products were not known. Different superscript letters denote significantly different percentages of farmers between years within a region at the 5% level (paired t-test). The data are from Q26, Q34 and Q40 of the questionnaire. | | | | | | | | | | | |

**Table S3: Insecticide treatment frequency index (TFI) – using t-tests**

|  | 2012 | 2013 | 2014 |
| --- | --- | --- | --- |
| Maize (Aquitaine) | 0.61^a^ | 0.65^a^ | 0.88^b^ |
| Maize (Aragon) | 0.03^a^ | 0.16^a^ | 0.24^b^ |
| Maize (Lombardy) | 1.18^a^ | 1.12^a^ | 1.10^a^ |
| OSR (Czech Republic) | 3.60^a^ | 3.86^b^ | 4.36^c^ |
| OSR (Eastern Germany) | 2.32^a^ | 2.40^a^ | 3.11^b^ |
| OSR (East of England) | 0.74^a^ | 0.72^a^ | 3.42^b^ |
| Sunflower (Andalusia) | 0.15^a^ | 0.10^a^ | 0.11^a^ |
| Sunflower (Northern Great Plain) | 0.23^a^ | 0.74^b^ | 0.85^b^ |
| The treatment frequency index (TFI) is the number of times each active substance is used, summed over all active substances. Different superscript letters indicate that values are significantly different at the 5% level (paired t-test). The data are from Q26, Q34 and Q40 of the questionnaire. | | | |

**Table S4: Yield (t/ha) – using t-tests**

|  | 2012 | 2013 | 2014 |
| --- | --- | --- | --- |
| Maize (Aquitaine) | 10.8^a^ | 10.0^b^ | 11.6^c^ |
| Maize (Aragon) | 13.0^a^ | 13.0^a^ | 13.0^a^ |
| Maize (Lombardy) | 10.9^a^ | 10.8^a^ | 10.0^a^ |
| OSR (Czech Republic) | 3.6^a^ | 4.0^b^ | 3.5^a^ |
| OSR (Eastern Germany) | 4.4^a^ | 4.7^b^ | 4.4^a^ |
| OSR (East of England) | 2.9^a^ | 2.8^a^ | 2.6^b^ |
| Sunflower (Andalusia) | 1.6^a^ | 1.5^a^ | 1.3^b^ |
| Sunflower (Northern Great Plain) | 4.8^a^ | 4.8^a^ | 4.9^a^ |
| The data are from Q10, Q11 and Q17 of the questionnaire. Different superscript letters indicate that values are significantly different at the 5% level (paired t-test). | | | |

**Table S5: Farmer perceptions of changes in wild beneficial insects (% of farmers)**

|  | *Maize* | | | *OSR* | | | *Sunflower* | |
| --- | --- | --- | --- | --- | --- | --- | --- | --- |
|  | Aquitaine | Aragon | Lombardy | Czech Republic | Eastern Germany | East of England | Andalusia | Northern Great Plain |
| Higher - similar - lower | 4 - 96 - 0 | 1 - 93 - 6 | 0 - 100 - 0 | 3 - 84 - 13 | 3 - 94 - 3 | 8 - 91 - 1 | 0 - 99 - 0 | 10 - 90 - 0 |
| The data are from Q53 of the questionnaire. | | | | | | | |  |

**Table S6: Yield (t/ha)**

|  | 2012 | 2013 | 2014 |
| --- | --- | --- | --- |
| Maize (Aquitaine) | 10.8^a^ | 10.0^b^ | 11.6^c^ |
| Maize (Aragon) | 13.0^a^ | 13.0^a^ | 13.0^a^ |
| Maize (Lombardy) | 10.9^a^ | 10.8^a^ | 10.0^b^ |
| OSR (Czech Republic) | 3.6^a^ | 4.0^b^ | 3.5^a^ |
| OSR (Eastern Germany) | 4.4^a^ | 4.7^b^ | 4.4^a^ |
| OSR (East of England) | 2.9^a^ | 2.8^a^ | 2.6^b^ |
| Sunflower (Andalusia) | 1.6^a^ | 1.5^a^ | 1.3^b^ |
| Sunflower (Northern Great Plain) | 4.8^a^ | 4.8^a^ | 4.9^a^ |
| The data are from Q10, Q11 and Q17 of the questionnaire. Different superscript letters indicate that values are significantly different at the 5% level (test of equality of matched pairs). | | | |

**Table S7: Per hectare valuation of removing CITF restrictions (% of farmers)**

|  | *Maize* | | | *OSR* | | | *Sunflower* | |
| --- | --- | --- | --- | --- | --- | --- | --- | --- |
|  | Aquitaine | Aragon | Lombardy | Czech Republic | Eastern Germany | East of England | Andalusia | Northern Great Plain |
| € 0 | 19 | 5 | 26 | 13 | 3 | 16 | 6 | 4 |
| € 1-5 | 13 | 12 | 16 | 1 | 14 | 27 | 55 | 9 |
| € 6-10 | 14 | 14 | 6 | 4 | 31 | 21 | 21 | 23 |
| € 11-15 | 3 | 10 | 4 | 3 | 13 | 3 | 16 | 36 |
| € 16-20 | 5 | 18 | 2 | 12 | 9 | 2 | 1 | 5 |
| € 21-25 | 2 | 1 | 0 | 4 | 4 | 1 | 1 | 2 |
| More than € 25 | 31 | 28 | 0 | 32 | 19 | 1 | 0 | 3 |
| Don't know | 13 | 12 | 20 | 24 | 5 | 29 | 1 | 11 |
| Don’t Answer | 0 | 0 | 24 | 7 | 2 | 0 | 0 | 6 |
| Median valuation | € 6-10 | € 16-20 | € 1-5 | € 21-25 | € 6-10 | € 1-5 | € 1-5 | € 11-15 |
| The data are from Q55 of the questionnaire. | | | | | | | | |

**Table S8: Valuation of CITF product characteristics (% of farmers)**

|  | *Maize* | | | *OSR* | | | *Sunflower* | |
| --- | --- | --- | --- | --- | --- | --- | --- | --- |
|  | Aquitaine | Aragon | Lombardy | Czech Republic | Eastern Germany | East of England | Andalusia | Northern Great Plain |
| Easy to use | 13 | 45 | 19 | 26 | 64 | 51 | 65 | 17 |
| Low cost | 14 | 28 | 9 | 13 | 32 | 47 | 27 | 30 |
| Easy to get | 0 | 25 | 2 | 2 | 12 | 47 | 27 | 11 |
| Effectiveness | 43 | 78 | 20 | 29 | 76 | 54 | 70 | 28 |
| No need for other treatments | 43 | 55 | 18 | 27 | 78 | 52 | 19 | 48 |
| Other characteristics | 3 | 11 | 1 | 17 | 10 | 1 | 0 | 1 |
| The data are from Q56 of the questionnaire, asking which characteristics of the restricted CITF products farmers value most. Multiple answers were allowed. | | | | | | | | |
