# Supplementary material for: The impact of restrictions on neonicotinoid and fipronil insecticides on pest management in maize, oilseed rape and sunflower in eight European Union regions
Source: Pest Manag Sci. 2017 Oct 13;74(1):88–99. doi: 10.1002/ps.4715 (PMC5765491; doi:10.1002/ps.4715)
Supplement: Supplementary file 3 — Questionnaire Round 2 [file PS-74-88-s002.docx]

**MAIN PHASE** N° interview: l__l__l__l

**SURVEY ON AGRONOMIC PRACTICES**

Date: l__l__l d l__l__l m l__l__l__l y Start time of interview: l__l__l h l__l__l m

**Q0: Please introduce your interviewer identification number** l__l__l__l__l__l

GENERAL QUESTIONNAIRE REMARKS:

1. Countries to be surveyed about **MAIZE** are **Spain, Italy and France.**
2. Countries to be surveyed about **SUNFLOWER** are **Hungary and Spain.**
3. Countries to be surveyed about **OSR** are **the UK, Germany and Czech Republic.**

The target crop is thus specific according to the country.

1. In Spain, Italy and France (maize), and Hungary (sunflower), the variable *Year* stands for:
   1. Year_D = 2014
   2. Year_D-1 = 2013
   3. Year_D-2 = 2012
   4. Year_D-3 = 2011
   5. Year_D-4 = 2010
2. In the UK, Germany and Czech Republic (OSR) the variable *Year* stands for:
   1. Year_D = 2014/15
   2. Year_D-1 = 2013/14
   3. Year_D-2 = 2012/13
   4. Year_D-3 = 2011/12
   5. Year D-4 = 2010/11
3. In Spain (Sunflower) the variable *Year* stands for:
   1. Year_D = 2015
   2. Year_D-1 = 2014
   3. Year_D-2 = 2013
   4. Year_D-3 = 2012
   5. Year_D-4 = 2011

Being the question number Qx, the variables corresponding to the year D are referred to as qx_1, D-1 year, will be named qx_2 and D-2 year are referred to as qx_3. For example for q6, the values of the year D will be recorded in the variable q6_1, D-1 year in the variable q6_2, etc.

For questions on economic values, each country should automatically select the currency, except for Spain which should provide a choice between Euros and pesetas. Confirmation of the resulting value will always be asked.

All measures of surface are recorded in hectares.

I.P.: Programming instructions.

I.E.: Instructions to the interviewer to be included in the question, uppercase or a different letter throughout the questionnaire. Instructions for programming and the interviewer also appear in red.

**Q1: TARGET CROP**

Single coded

MAIZE 1

SUNFLOWER 2

RAPESEED 3

**Q2: TARGET COUNTRY**

Single coded

Czech Republic 1 

France 2 

Germany 3 

Hungary 4 

Italy 5 

Spain 6 

United Kingdom 7 

**Q3: REGION**

SPAIN

Ebro Valley 1 

Cádiz *(Sunflower)* 4 

**Q4: MUNICIPALITY**

Please insert the name of the MUNICIPALITY where the farm is located

|  |
| --- |

**INTERVIEWER, READ OUT LOUD**

The Institute for Prospective Technological Studies, part of the Joint Research Centre of the European Commission (JRC-IPTS), is engaged in an important investigation of the European agricultural sector. Among the Member States of the European Union, [SPAIN/HUNGARY/ITALY/SPAIN/GERMANY/UK/CZECH REPUBLIC] is a key country for [MAIZE/OSR/SUNFLOWER] production. To carry out this investigation, JRC-IPTS entrusted to TNS Demoscopia a survey among farmers cultivating [MAIZE/OSR/SUNFLOWER].

TNS, together with the relevant regional agrarian offices, cooperatives, agricultural associations, etc., has determined that your municipality is among the most important within the country for the purpose of this investigation. For this reason you have been selected for the study.

Therefore, we request your kind collaboration through a personal interview, which can provide us with important information related to the cultivation of [MAIZE/OSR/SUNFLOWER] in your region.

This study is dedicated to collect and analyze data and has no commercial purpose. Our Institute guarantees that all information that you provide will be treated strictly confidentially and only for the purpose of research. Your answers will be analyzed in a way so that the answers cannot be associated with one person in particular.

We are at your disposal if you want to learn more about this project. Thank you in advance for your availability.

Q5: What is your role in the farm?

Single coded

Head of the Farm / household 1 

General Manager 2 

Section head 3 

Field worker 4  SCREEN OUT

Other (specify) ______________ _7  SCREEN OUT IF NOT RESPONSIBLE FOR SEED CHOICE OR CROP MAINTENANCE

Q6: What was your TOTAL FARM Utilized Agricultural Area in Year_D, Year_D-1, Year_D-2, Year_D-3, and Year_D-4? Please consider the sum of all the cultivated plots, rented, owned, etc. but excluding lakes forests and rivers.

Multi coded

|  |  |  | Don't Answer  (DO NOT READ) |
| --- | --- | --- | --- |
| 1 | Year_D | \|___\|___\|___\|___\| ha |  |
| 2 | Year_D-1 | \|___\|___\|___\|___\| ha |  |
| 3 | Year_D-2 | \|___\|___\|___\|___\| ha |  |
| 4 | Year_D-3 | \|___\|___\|___\|___\| ha |  |
| 5 | Year_D-4 | \|___\|___\|___\|___\| ha |  |

Q7: Have you cultivated [MAIZE/OSR/SUNFLOWER] in Year_D?

Single coded

Yes 1  GO TO Q8

No 2  GO TO Q10

Don't know (DO NOT READ) 8  SCREEN OUT

Don't answer (DO NOT READ) 9  SCREEN OUT

Q8. What surface of [MAIZE/OSR/SUNFLOWER] have you cultivated in Year_D? _______________________ GO TO Q9.

Q9. [ONLY MAIZE] what type of [MAIZE] have you grown in Year_D:

1. Grain [MAIZE] |___|___|___|___| ha
2. Green [MAIZE] SCREEN OUT
3. Both (In this case we will ask all the following questions just for Grain [MAIZE])

(I.P. surface for Grain Maize is required in this case).

Q10: Have you cultivated [MAIZE/OSR/SUNFLOWER] in any of the Year_D-1 or Year_D-2? If this is the case, could you please state the surface for each of the years?

Single coded for each year;

|  |  | Year_D-1 | Q10.a. SURFACE Cultivated. (ha) | Year_D-2 | Q10.b. SURFACE Cultivated.(ha) |  |
| --- | --- | --- | --- | --- | --- | --- |
| 1 | Yes |  | \|___\|___\|___\|___\|ha |  | \|___\|___\|___\|___\| ha | IF ALL YEARS MARKED GO TO Q11b |
| 2 | No |  | -- |  | -- | IF ANY YEAR MARKED GO TO Q11a |
| 8 | Don't know  (DO NOT READ) |  | -- |  | -- | IF ALL MARKED SCREEN OUT |
| 9 | Don't answer  (DO NOT READ) |  | -- |  | -- | IF ALL MARKED SCREEN OUT |

Q11a: What was the last year before D-2 in which you cultivated [MAIZE/OSR/SUNFLOWER], and what surface? (multiple answers possible) (IP: in case the answer is D-3 or D-4; also use that year for the rest of the questionnaire instead of D-1 or D-2, according to these criteria the possible combinations are: D/D-1/D-2 – D/D-2/D-3 - D/D-3/D-4 – D/D-1/D-3 – D/D-1/D-4 – D/D-2/D-4).

I have never before cultivated [*MAIZE/OSR/SUNFLOWER*] 1  SCREEN OUT if Q10=2

D-3 .......... 2  Surface (Ha.) _____________

D-4 .......... 3  Surface (Ha.) _____________

Other, (specify) _____4  SCREEN OUT

Q11b; Have you used any of the following products in Year_D, Year_D-X and/or Year_D-Y in [MAIZE/OSR/SUNFLOWER]?

(READ OUT) Please mark all seed treatments you have used in any of these years.

Do not consider GM [MAIZE]; *(<*- *This comment is only for [MAIZE] in Spain)*
Multiple answers coded

Maize:

(ES)

Regent 10 

Poncho 11 

Escocet 12 

Picus 13 

Seedoprid 14 

Cruiser 15 

(FRA)

Cruiser 15 

Cheyenne. 17 

(ITA)

Gaucho 18 

Cruiser 15 

Nuprid 19 

Poncho 11 

Santana 20 

Sunflower

(HU)

Gaucho 18 

Cruiser 15 

(ES)

Regent 10 

Cruiser 15 

OSR

(GER)

Antarc 22 

Chinook 23 

Cruiser 15 

Elado 24 

Modesto 25 

(UK)

Chinook 23 

Cruiser 15 

Modesto 25 

(CZ)

Cruiser 15 

Elado 24 

Modesto 25 

*None of them 97  SCREEN OUT*

*Don't know (DO NOT READ) 98  SCREEN OUT*

*Don't Answer (DO NOT READ) 99  SCREEN OUT*

Q11c. What is the main reason why you have decided not to cultivate [MAIZE/OSR/SUNFLOWER] in year_D?.[I.P.: SINGLE ANSWER. For those with Q7=2]

Normal crop rotation 1  SCREEN OUT

[list of products] not available for seed treatment 2  [skip questions for Year D, and Q46 to Q53]

[list of products] not available for soil or foliar treatment3  [skip questions for Year D, and Q46 to Q53]

Other reason (specify) (DO NOT READ) 4  SCREEN OUT

**Q12: Please indicate all insecticide seed treatment products of [MAIZE/OSR/SUNFLOWER] you used in year_D, D-X and D-Y.** In case you used more than one type of seed treatment, or untreated seed, for different parts of your total farm seed, please indicate them separately. In addition, indicate the hectares, quantity, and cost of the seed corresponding to each seed treatment product (or non-treated seed). (I.E. there is larger number of possible seed treatments a farmer could have used than the list on Q11b contains. All seed treatments that a farmer has used, whether in the list of Q11b or not, should be recorded, as well as the amount of seed untreated, if any.). Multi coded. [I.E.: Please write 998 for don’t know and 999 for don’t answer] **(I.E.:** “Leave blank if the respondent do not have any untreated seeds”).

Year_D

| **a. Name of seed treatment**  (active substance and/or brand name) | **b. Hectares** | **c. Quantity (Kg./Tn./Quintals)** | **d. Cost**  *(I.P.: confirm the currency)* | **e. Unit used** |
| --- | --- | --- | --- | --- |
|  | \|___\|___\|___\| **ha** | \|___\|___\|___\|___\| | \|___\|___\|___\|___\| |  PER HECTARE   TOTAL |
|  | \|___\|___\|___\| **ha** | \|___\|___\|___\|___\| | \|___\|___\|___\|___\| |  PER HECTARE   TOTAL |
|  | \|___\|___\|___\| **ha** | \|___\|___\|___\|___\| | \|___\|___\|___\|___\| |  PER HECTARE   TOTAL |
| Not treated | \|___\|___\|___\| **ha** | \|___\|___\|___\|___\| | \|___\|___\|___\|___\| |  PER HECTARE   TOTAL |

Year_D-X

| **a. Name of seed treatment**  (active substance and/or brand name) | **b. Hectares** | **c. Quantity (Kg./Tn./Quintals)** | **d. Cost**  *(I.P.: confirm the currency)* | **e. Unit used** |
| --- | --- | --- | --- | --- |
|  | \|___\|___\|___\| **ha** | \|___\|___\|___\|___\| | \|___\|___\|___\|___\| |  PER HECTARE   TOTAL |
|  | \|___\|___\|___\| **ha** | \|___\|___\|___\|___\| | \|___\|___\|___\|___\| |  PER HECTARE   TOTAL |
|  | \|___\|___\|___\| **ha** | \|___\|___\|___\|___\| | \|___\|___\|___\|___\| |  PER HECTARE   TOTAL |
| Not treated | \|___\|___\|___\| **ha** | \|___\|___\|___\|___\| | \|___\|___\|___\|___\| |  PER HECTARE   TOTAL |

Year_D-Y

| **a. Name of seed treatment**  (active substance and/or brand name) | **b. Hectares** | **c. Quantity (Kg./Tn./Quintals)** | **d. Cost**  *(I.P.: confirm the currency)* | **e. Unit used** |
| --- | --- | --- | --- | --- |
|  | \|___\|___\|___\| **ha** | \|___\|___\|___\|___\| | \|___\|___\|___\|___\| |  PER HECTARE TOTAL |
|  | \|___\|___\|___\| **ha** | \|___\|___\|___\|___\| | \|___\|___\|___\|___\| |  PER HECTARE TOTAL |
|  | \|___\|___\|___\| **ha** | \|___\|___\|___\|___\| | \|___\|___\|___\|___\| |  PER HECTARE TOTAL |
| Not treated | \|___\|___\|___\| **ha** | \|___\|___\|___\|___\| | \|___\|___\|___\|___\| |  PER HECTARE TOTAL |

**Q12a: Before Year_D, were you able to buy [MAIZE/OSR/SUNFLOWER] seed that were not treated with** (I.P.: Include the list of products for the crop/country, used in question 11b)?

Yes **1 **

No **2 **

Don’t know **(DO NOT READ) 8 **

Don’t Answer **(DO NOT READ) 9 **

**Q13: Do you practice rotation in your [MAIZE/OSR/SUNFLOWER]?**

Single coded

| Yes 1  | **Q13a: If yes, please specify the surface you normally manage using rotation for [MAIZE/OSR/SUNFLOWER].**  \|___\|___\|___\|___\| **ha** |
| --- | --- |
| No 2  | [I.P.: Q13<q8] |

**Q14:** **[I.P.: SKIP IF Q13=NO]. Which of the following crops normally precede [MAIZE/OSR/SUNFLOWER]?**

Single coded; Just 3 main answers in terms of cultivated surface.

(READ OUT). Rotate 1-7.

*(I.P.: Max. of 3 responses, but 1 or 2 are allowed).*

Maize 1 

Potato 2 
Sunflower 3 

Legumes 4 
Sugar beet 5 

Winter Wheat 6 

Winter Barley 7 

Other, (specify) _______________8 

Don't know 9 

**Q15: How many hectares of the [MAIZE/OSR/SUNFLOWER] total cultivated surface were irrigated in year_D, year_D-X, and year D-Y? Please also state the cost of irrigation in each year.**

[I.E.: Interviewer, please state the number of hectares]

(READ OUT) Please select all the years that apply and state the surface irrigated. Multi coded

| 1 | Year_D | \|___\|___\|___\|Ha. | \|___\|___\|___\| **€** |  PER HECTARE  TOTAL |
| --- | --- | --- | --- | --- |
| 2 | Year_D-X | \|___\|___\|___\|Ha. | \|___\|___\|___\| **€** |  PER HECTARE  TOTAL |
| 3 | Year_D-Y | \|___\|___\|___\|Ha. | \|___\|___\|___\| **€** |  PER HECTARE  TOTAL |

I.P.:/I.E.: Do not to include the years in which not cultivated [MAIZE/OSR/SUNFLOWER], according to Q7.

998 if the respondent Don't Know. 999 if the respondent Don't Answer. If the land is not irrigated please put 0. (I.E.: The cost should be automatically switched to “o”).

**Q16: Did you hire any seasonal or permanent workers for the production of [MAIZE/OSR/SUNFLOWER] in the Year_D, and in Year_D-X and Year_D-Y)? If yes, how many full days did hired workers work in [MAIZE/OSR/SUNFLOWER] production and how much did you pay them?**

| 1 | Year_D | \|___\|___\|___\|  Total workers | \|___\|___\|___\| days/year | \|___\|___\|___\| **€** |  PER day   TOTAL (full season) |
| --- | --- | --- | --- | --- | --- |
| 2 | Year_D-X | \|___\|___\|___\|  Total workers | \|___\|___\|___\| days/year | \|___\|___\|___\| **€** |  PER day   TOTAL (full season) |
| 3 | Year_D-Y | \|___\|___\|___\|  Total workers | \|___\|___\|___\| days/year | \|___\|___\|___\| **€** |  PER day   TOTAL (full season) |

**Q17: How much [MAIZE/OSR/SUNFLOWER] did you harvest on your farm in the Year_D, and in (Year_D-X – Year_D-Y)?**

|  |  | Please write clear figures. | Don't Know (DO NOT READ) | Don't Answer (DO NOT READ) | **Q17a:** (DO NOT READ)  **What unit did the interviewee use?**  Single coded | **Q17b:** (DO NOT READ)  **What unit did the interviewee use?**  Single coded |
| --- | --- | --- | --- | --- | --- | --- |
| 1 | Year_D | \|___\|___\|___\| |  |  | Kgs. 1   Tones 2   Quintals 3  | Total. 1   Per Hectare 2  |
| 2 | Year_D-X | \|___\|___\|___\| |  |  |  |  |
| 3 | Year_D-Y | \|___\|___\|___\| |  |  |  |  |

**Q18: What price did/would you receive for your [MAIZE/OSR/SUNFLOWER] in...?**

If you cannot indicate an exact price, please indicate your best estimate.

|  |  | Please write clear figures. | Don't Know (DO NOT READ) | Don't Answer (DO NOT READ) | [DO NOT READ, NOTE] | |
| --- | --- | --- | --- | --- | --- | --- |
|  |  |  |  |  | Q18.a: What unit did the interviewee use?   1. Euros [*Spain*] 2. Pesetas [*Spain*] 3. Czech Crown 4. Forints 5. Pounds | Q18.b: What unit did the interviewee use?   1. Kgs. 2. Tones 3. Quintals. |
| 1 | Year_D | \|___\|___\|___\| |  |  | \|___\| | \|___\| |
| 2 | Year_D-X | \|___\|___\|___\| |  |  | \|___\| | \|___\| |
| 3 | Year_D-Y | \|___\|___\|___\| |  |  | \|___\| | \|___\| |

**Q19: Did you use any of the following fertilizers in [MAIZE/OSR/SUNFLOWER] production?**

|  |  | Nitrogen (N) | Phosphorus(P) | Potassium (K) | Manure | None |
| --- | --- | --- | --- | --- | --- | --- |
| 1 | Year_D |  |  |  |  |  |
| 2 | Year_D-X |  |  |  |  |  |
| 3 | Year_D-Y |  |  |  |  |  |

**Q20: [I.P.: Only if any product selected in Q19] How much money did you spent on fertilizers in [MAIZE/OSR/SUNFLOWER] production in the Year_D and in the previous years?**

Please provide a GLOBAL figure taking into account the FULL SEASON

|  |  | Expenditure |  | Don't Know  (DO NOT READ) | Don't Answer/Do not apply  (DO NOT READ) |
| --- | --- | --- | --- | --- | --- |
| 1 | Year_D | \|___\|___\|___\|___\| € |  PER HECTARE   TOTAL |  |  |
| 2 | Year_D-X | \|___\|___\|___\|___\| € |  PER HECTARE   TOTAL |  |  |
| 3 | Year_D-Y | \|___\|___\|___\|___\| € |  PER HECTARE   TOTAL |  |  |

**Q21: Were you part of an Integrated Pest Management (IPM) or organic farming program or certification scheme that includes your [MAIZE/OSR/SUNFLOWER] production in the last or any of the previous years?** (READ OUT);

|  |  | Year_D | Year_D-X | Year_D-Y |
| --- | --- | --- | --- | --- |
| 1 | Integrated Pest Management (IPM) |  |  |  |
| 2 | Organic farming certification |  |  |  |
| 3 | None of the Above | **** | **** | **** |
| 8 | Don't Know (DO NOT READ) | **** | **** | **** |
| 9 | Don't answer (DO NOT READ) | **** | **** | **** |

**Q22: Which of the following types of soil management did you use in your [MAIZE/OSR/SUNFLOWER] culture?**

(READ OUT); Also add the surface of [MAIZE/OSR/SUNFLOWER] area with all options used.

Multi coded

|  |  | Year_D  (I.P.: ∑Q22 <=Q9)  (for maíze) | Year_D-X  (I.P.: ∑Q22 <=Q8)  (for sunflower/OSR) | Year_D-Y  (I.P.: ∑Q22 <=Q8)  (for sunflower/OSR) |
| --- | --- | --- | --- | --- |
| 1 | Conventional tillage |  \|___\|___\|___\|___\| ha |  \|___\|___\|___\|___\|ha |  \|___\|___\|___\|___\|ha |
| 2 | Conservation/minimum tillage |  \|___\|___\|___\|___\| ha |  \|___\|___\|___\|___\|ha |  \|___\|___\|___\|___\|ha |
| 3 | No tillage |  \|___\|___\|___\|___\| ha |  \|___\|___\|___\|___\|ha |  \|___\|___\|___\|___\|ha |
| 4 | Other (specify)________ |  \|___\|___\|___\|___\| ha |  \|___\|___\|___\|___\|ha |  \|___\|___\|___\|___\|ha |
| 8 | Don't Know  (DO NOT READ) | **** | **** | **** |
| 9 | Don't answer  (DO NOT READ) | **** | **** | **** |

| **B1 : POST RESTRICTION PRACTICES** |
| --- |

**Q23: [MAIZE/SUNFLOWER/OSR] In Year_D, how many applications of PHYTOSANITARIAN PRODUCTS did you do in your culture?**

**Please consider the total number of times you entered your crop for treating in Year_D. Do not consider seed treatments.**

**|___|___| Times**

**Q23A [I.P.: New, and only for OSR] And more specifically, in Year_D, in how many of the times, you just mentioned, you entered your crop for treating, did the applications contain HERBICIDES, in how many did they contain INSECTICIDES and in how many did they contain FUNGICIDES?.**

(Interviewer: Note and explain to farmers that some applications may include more than one phytosanitarian product).

(I.P. Q23 = or < than ∑Q23A).

*Do not consider seed treatments.*

**|___|___| Number of Herbicide Applications.**

**|___|___| Number of Insecticide Applications.**

**|___|___| Number of Fungicide Applications.**

Interviewer, read out loud: **Now we are going to speak about each one of these applications. [FOR OSR only –…including at least one insecticide].**

Interviewer, read Q25 to Q30 for each application. Note down information for up to 3 times for each question, according to the number of times given in Q23, writing down the code or verbatim on each appropriate cell

**Q24: How much did you spend on plant protection products for your [MAIZE/OSR/SUNFLOWER] Year_D?** Please provide a GLOBAL figure taking into account the FULL SEASON. Please note that seed treatments are not included.

|  |  |  |  | Don't Know (DO NOT READ) | Don't Answer/Do not apply (DO NOT READ) |
| --- | --- | --- | --- | --- | --- |
| 1 | Year_D | \|___\|___\|___\|___\| € |  PER HECTARE   TOTAL |  |  |

**According to your response, in the following questions we will be talking about your use of plant protection products in Year_D taking into account each time you entered your crop [FOR OSR only – to treat including at least one insecticide] even if you used two or more products at the same time.**

**I.E.: Please complete all the questions about an application before going to the questions about the following application.**

**I.E.: In case that the number of applications exceed the maximum allowed, (max. of 3 applications), please include the active substance and the pest treated used in the forth and above applications in the third application and note this fact in the observation of the questionnaire, with details.**

|  | **Year_D** | | |
| --- | --- | --- | --- |
| [I.E.: ASK AS MANY APPLICATIONS AS INDICATED IN Q23]. Min 1 \| Max 3 | **App. 1** | **App.2** | **App.3** |
| **Q25: [I.P.NOT FOR OSR] Did this application consist of insecticides, fungicides and/or herbicides?**  Multi coded. ASK Q26A, B AND/OR C WHEN APPROPIATE  Yes, Herbicides (ASK Q26A HERBICIDES)  Yes, Insecticides (ASK Q26B INSECTICIDES)  Yes, Fungicides (ASK Q26C FUNGICIDES) | 1   2   3  | 1   2   3  | 1   2   3  |
| **Q26: Which of the following products (active substances) were contained in this application? Please indicate the active substance and the brand name belonging to the active substance. If you do not know the active substance you used, please indicate at least the brand name.**  [READ OUT depending on the answer given in Q25; All the OPTIONS included] Multi coded. SHOWCARD  A. HERBICIDES [I.P.NOT FOR OSR]  Glyphosate 1  Terbuthylazine 2  Other herbicide (specify) 97  Don't know (DO NOT READ) 98 Don't Answer (DO NOT READ) 99  **B. INSECTICIDES**  MAIZE  Abamectine 3  Ethoprophos 4  Alfa cypermethrin 5  Cyfluthrin 6  Hexythiazox 7  Chlorpyrifos 8  Chlorpyrifos Methyl 9  Deltamethrin 10  Lambda Cyhalothrin 11  Tefluthrin 12  SUNFLOWER  Deltamethrin 13  Chlorpyrifos 14  Pirimicarb 15  Teflutrhin 16  OSR  Cypermethrin 17  Deltamethrin 18  Esfenvalerate 19  Lambda Cyhalothrin 20  Pirimicarb 21  Other insecticide (specify) 91  Don't know active substance (NOT READ) 92  Don't Answer (NOT READ) 93  **C. FUNGICIDES [I.P.NOT FOR OSR]**  **MAIZE**  Cyproconazole Epoxiconazole 22  Sulphur 23  Mancozeb 24  Methyl Thiphanate 25  **SUNFLOWER**  Fenpropimorph 26  Mancozeb 27  **OSR**  Mancozeb 27  Methyl Thiphanate 29  Propiconazole 30  Tebuconazole 31  Other fungicide (specify) 94  Don't know (DO NOT READ) 95  Don't Answer (DO NOT READ) 96 | 1 ______  2 ______  97 ______  98   99   3 ______  4 ______  5 ______  6 ______  7 ______  8 ______  9 ______  10 ______  11 ______  12 ______  13 ______  14 ______  15 ______  16 ______  17 ______  18 ______  19 ______  20 ______  21 ______  91 ______  92   93   22 ______  23 ______  24 ______  25 ______  26 ______  27 ______  27 ______  29 ______  30 ______  31 ______  94 ______  95   96  | 1 ______  2 ______  97 ______  98   99   3 ______  4 ______  5 ______  6 ______  7 ______  8 ______  9 ______  10 ______  11 ______  12 ______  13 ______  14 ______  15 ______  16 ______  17 ______  18 ______  19 ______  20 ______  21 ______  91 ______  92   93   22 ______  23 ______  24 ______  25 ______  26 ______  27 ______  27 ______  29 ______  30 ______  31 ______  94 ______  95   96  | 1 ______  2 ______  97 ______  98   99   3 ______  4 ______  5 ______  6 ______  7 ______  8 ______  9 ______  10 ______  11 ______  12 ______  13 ______  14 ______  15 ______  16 ______  17 ______  18 ______  19 ______  20 ______  21 ______  91 ______  92   93   22 ______  23 ______  24 ______  25 ______  26 ______  27 ______  27 ______  29 ______  30 ______  31 ______  94 ______  95   96  |
| **Q27: What type of treatment did you use in this application?**  Single coded  Soil treatment 1  Foliar treatment 2  Other (specify) 4  Don't know (DO NOT READ) 8  Don't Answer (DO NOT READ) 9 | 1   2   4   8   9  | 1   2   4   8   9  | 1   2   4   8   9  |
| **Q28: What was the crop stage when the application took place?** READ OUT, single code  Before Sowing 1  During Sowing 2  After sowing/pre-emergence 3  Vegetative stage 4  Flowering stage 5  Fructification stage 6  Other (DO NOT READ) 8  Don't Answer (DO NOT READ) 9 | 1   2   3   4   5   6   8   9  | 1   2   3   4   5   6   8   9  | 1   2   3   4   5   6   8   9  |

| **Q29: What was the pest treated with this application?**  (I.E.: SHOWCARD AND READ OUT LOUD. Multi coded)/**I.P.: IF herbicide code 1, “Weeds”, should automatically be assigned)**  (MAIZE cod.2-7, & 27)  Corn Borer (ECB/WCB) 2  Cutworms 3  Wireworms 4  Lepidopteran leaf feeders 5  Leafhoppers 6  Insect borne viruses 7  Diabrotica virgifera 26  (OSR cod. 4-26; skip 5-7 & 21)  Wireworms 4  Alternaria 8  Peach/Potate Aphid 9  Brassica pod midge 10  Cabagge aphid 11  Cabagge flea beetle 12  Cabagge root fly 13  Cabagge seed weevil 14  Cabagge stem flea beetle 15  Cabagge stem weevil 16  Cylindrosporium 17  Pod midge 20  Pollen beetles 21  Rape stem weevil 23  Rape winter stem weevil 24  Sclerotinia 25  Phoma stem canker 27  (SUNFLOWER cod.3-39 skip 5-27)  Cutworms 3  False wireworms 28  Heliothis / Helicoverpa spp. 29  Root and stem charcoal rot 30  Sunflower broomrape 31  Sunflower downy mildew 32  Sunflower moth 33  Verticilosis 34  Weevils, 35  White grubs, 36  White rot 37  Wireworms 4  Gray rot 38  Sunflower rust 39  Grey Spot 40  Aphids 41  None of the above 96  Other (specify) 97  Don't know (DO NOT READ) 98  Don't answer (DO NOT READ) 99 | 2   3   4   5   6   7   27   4   8   9   10   11   12   13   14   15   16   17   20   22   23   24   25   27   3   28   29   30   31   32   33   34   35   36   37   4   38   39   40   41   96   97 _______  98   99  | 2   3   4   5   6   7   27   4   8   9   10   11   12   13   14   15   16   17   20   22   23   24   25   27   3   28   29   30   31   32   33   34   35   36   37   4   38   39   40   41   96   97 _______  98   99  | 2   3   4   5   6   7   27   4   8   9   10   11   12   13   14   15   16   17   20   22   23   24   25   27   3   28   29   30   31   32   33   34   35   36   37   4   38   39   40   41   96   97 _______  98   99  |
| --- | --- | --- | --- |
| **Q30: What was the cost of the treatment?**  (I.P.; control currency unit)  (I.E.: Cost of application including product and machinery, handwork, etc.).  Don't know (DO NOT READ) 998  Don't Answer (DO NOT READ) 999 | Per Hectare   Total.   **\|__\|__\|__\|** € | Per Hectare   Total.   **\|__\|__\|__\|** € | Per Hectare   Total.   **\|___\|___\|___\|** € |

| **B2: PRE RESTRICTION PRACTICES** |
| --- |

**In the following questions we will be talking about the previous years you cultivated [MAIZE/OSR/SUNFLOWER], i.e. Year_D-X AND/OR Year_D-Y**

**Q31: [MAIZE/SUNFLOWER/OSR] In the Years_D-X and D-Y, how many APPLICATIONS OF PHYTOSANITARIAN PRODUCTS did you do in your culture?**

**Please consider the total number of times you entered your crop for treating in the Year. Do not consider seed treatments.**

**|___|___| Times D-X**

**|___|___| Times D-Y**

**Q31A [I.P.: New, and only for OSR] And more specifically, in Year_D-X and in Year D-Y, in how many of the times, you just mentioned, you entered your crop for treating, did the applications contain HERBICIDES, in how many did they contain INSECTICIDES and in how many did they contain FUNGICIDES?.** *Do not consider seed treatments.*

(Interviewer: Note and explain to farmers that some applications may include more than one phytosanitarian product).

(I.P. Q31 = or < than ∑Q31A, D-X or D-Y).

| **(Year D-X).** | **(Year D-Y).** |
| --- | --- |
| **\|___\|___\| Number of Herbicide Applications.** | **\|___\|___\| Number of Herbicide Applications.** |
| **\|___\|___\| Number of Insecticide Applications.** | **\|___\|___\| Number of Insecticide Applications.** |
| **\|___\|___\| Number of Fungicide Applications.** | **\|___\|___\| Number of Fungicide Applications.** |

**Q32: How much did you spend on plant protection products for your [MAIZE/OSR/SUNFLOWER], in Year_D-X and year_D-Y?** Please provide a GLOBAL figure taking into account the FULL SEASON. Please note that seed treatments are not included.

|  |  |  |  | Don't Know (DO NOT READ) | Don't Answer/Do not apply (DO NOT READ) |
| --- | --- | --- | --- | --- | --- |
| 1 | Year_D-X | \|___\|___\|___\|___\| € |  PER HECTARE   TOTAL |  |  |
| 2 | Year_D-Y | \|___\|___\|___\|___\| € |  PER HECTARE   TOTAL |  |  |

Interviewer, read out loud: **Now we are going to speak about each one of these applications [FOR OSR only – Including at least one insecticide].**

Interviewer, read Q33 to Q38 for each application. Note down information for up to 3 times for each question, according to the number of times given in Q31, writing down the code or verbatim on each appropriate cell.

**According to your response, in the following questions we will be talking about your use of PLANT PROTECTION PRODUCTS / INSECTICIDES in the Year_D-X and Year_D-Y taking into account each time you entered your crop [FOR OSR only – to treat including at least one insecticide] even if you used two or more products at the same time.**

I.E.: Please complete all the questions about an application before going to the questions about the following application.

**I.E.: In case that the number of applications exceed the maximum allowed, (max. of 3 applications), please include the active substance and the pest treated used in the forth and above applications in the third application and note this fact in the observation of the questionnaire, with details.**

|  | **Year_D-X** | | |
| --- | --- | --- | --- |
| [I.E.: ASK AS MANY APPLICATIONS AS INDICATED IN Q23] Min 1 \| Max 3. | **App. 1** | **App. 2** | **App. 3** |
| **Q33: [I.P.NOT FOR OSR] Did this application consist of insecticides, fungicides and/or herbicides?** I.P.: ASK Q40A, B AND/OR C WHEN APPROPIATE  Yes, Herbicides (ASK Q34A HERBICIDES)  Yes, Insecticides (ASK Q34B INSECTICIDES)  Yes, Fungicides (ASK Q34C FUNGICIDES) | 1   2   3  | 1   2   3  | 1   2   3  |
| **Q34: Which of the following products (active substances) were contained in this application? Please indicate the active substance and the brand name belonging to the active substance. If you do not know the active substance you used, please indicate at least the brand name.**  [READ OUT depending on the answer given in Q33; All the OPTIONS included] Multi coded. SHOWCARD  A. HERBICIDES [I.P.NOT FOR OSR]  Glyphosate 1  Terbuthylazine 2  Other herbicide (specify) 97  Don't know (DO NOT READ) 98 Don't Answer (DO NOT READ) 99  **B. INSECTICIDES**  MAIZE  Abamectine 3  Ethoprophos 4  Alfa cypermethrin 5  Cyfluthrin 6  Hexythiazox 7  Chlorpyrifos 8  Chlorpyrifos Methyl 9  Deltamethrin 10  Lambda Cyhalothrin 11  Tefluthrin 12  SUNFLOWER  Deltamethrin 13  Chlorpyrifos 14  Pirimicarb 15  Teflutrhin 16  OSR  Cypermethrin 17  Deltamethrin 18  Esfenvalerate 19  Lambda Cyhalothrin 20  Pirimicarb 21  Other insecticide (specify) 91  Don't know active substance (NOT READ) 92  Don't Answer (NOT READ) 93  **C. FUNGICIDES [I.P.NOT FOR OSR]**  **MAIZE**  Cyproconazole Epoxiconazole 22  Sulphur 23  Mancozeb 24  Methyl Thiphanate 25  **SUNFLOWER**  Fenpropimorph 26  Mancozeb 27  **OSR**  Mancozeb 27  Methyl Thiphanate 29  Propiconazole 30  Tebuconazole 31  Other fungicide (specify) 94  Don't know (DO NOT READ) 95  Don't Answer (DO NOT READ) 96 | 1 ______  2 ______  97 ______  98   99   3 ______  4 ______  5 ______  6 ______  7 ______  8 ______  9 ______  10 ______  11 ______  12 ______  13 ______  14 ______  15 ______  16 ______  17 ______  18 ______  19 ______  20 ______  21 ______  91 ______  92   93   22 ______  23 ______  24 ______  25 ______  26 ______  27 ______  27 ______  29 ______  30 ______  31 ______  94 ______  95   96  | 1 ______  2 ______  97 ______  98   99   3 ______  4 ______  5 ______  6 ______  7 ______  8 ______  9 ______  10 ______  11 ______  12 ______  13 ______  14 ______  15 ______  16 ______  17 ______  18 ______  19 ______  20 ______  21 ______  91 ______  92   93   22 ______  23 ______  24 ______  25 ______  26 ______  27 ______  27 ______  29 ______  30 ______  31 ______  94 ______  95   96  | 1 ______  2 ______  97 ______  98   99   3 ______  4 ______  5 ______  6 ______  7 ______  8 ______  9 ______  10 ______  11 ______  12 ______  13 ______  14 ______  15 ______  16 ______  17 ______  18 ______  19 ______  20 ______  21 ______  91 ______  92   93   22 ______  23 ______  24 ______  25 ______  26 ______  27 ______  27 ______  29 ______  30 ______  31 ______  94 ______  95   96  |
| **Q35: What type of treatment did you use in this application?**  Single coded  Soil treatment 1  Foliar treatment 2  Other (specify) 4  Don't know (DO NOT READ) 8  Don't Answer (DO NOT READ) 9 | 1   2   4   8   9  | 1   2   4   8   9  | 1   2   4   8   9  |
| **Q36: What was the crop stage when the application took place? (**READ OUT, single code)  Before Sowing 1  During Sowing 2  After sowing/pre-emergence 3  Vegetative stage 4  Flowering stage 5  Fructification stage 6  Other (DO NOT READ) 8  Don't Answer (DO NOT READ) 9 | 1   2   4   5   6   8   9  | 1   2   4   5   6   8   9  | 1   2   4   5   6   8   9  |

| **Q37: What was the pest treated with this application?**(I.E.: SHOWCARD AND READ OUT LOUD. Multi coded. I.P.: IF herbicide code 1, “Weeds”, should automatically be assigned) [I.P. OSR would not be asked about weeds].  (MAIZE cod.2-7, & 27)  Corn Borer (ECB/WCB) 2  Cutworms 3  Wireworms 4  Lepidopteran leaf feeders 5  Leafhoppers 6  Insect borne viruses 7  Diabrotica virgifera 26  (OSR cod. 4-26; skip 5-7 & 21)  Wireworms 4  Alternaria 8  Peach/Potate Aphid 9  Brassica pod midge 10  Cabagge aphid 11  Cabagge flea beetle 12  Cabagge root fly 13  Cabagge seed weevil 14  Cabagge stem flea beetle 15  Cabagge stem weevil 16  Cylindrosporium 17  Pod midge 20  Pollen beetles 21  Rape stem weevil 23  Rape winter stem weevil 24  Sclerotinia 25  Phoma stem canker 27  (SUNFLOWER cod.3-39 skip 5-27)  Cutworms 3  False wireworms 28  Heliothis / Helicoverpa spp. 29  Root and stem charcoal rot 30  Sunflower broomrape 31  Sunflower downy mildew 32  Sunflower moth 33  Verticilosis 34  Weevils, 35  White grubs, 36  White rot 37  Wireworms 4  Gray rot 38  Sunflower rust 39  Grey Spot 40  Aphids 41  None of the above 96  Other (specify) 97  Don't know (DO NOT READ) 98  Don't answer (DO NOT READ) 99 | 2   3   4   5   6   7   27   4   8   9   10   11   12   13   14   15   16   17   20   22   23   24   25   27   3   28   29   30   31   32   33   34   35   36   37   4   38   39   40   41   96   97 _______  98   99  | 2   3   4   5   6   7   27   4   8   9   10   11   12   13   14   15   16   17   20   22   23   24   25   27   3   28   29   30   31   32   33   34   35   36   37   4   38   39   40   41   96   97 _______  98   99  | 2   3   4   5   6   7   27   4   8   9   10   11   12   13   14   15   16   17   20   22   23   24   25   27   3   28   29   30   31   32   33   34   35   36   37   4   38   39   40   41   96   97 _______  98   99  |
| --- | --- | --- | --- |
| **Q38: What was the cost of the treatment?**  (I.P.; control currency unit)  (I.E.: Cost of application including product and machinery, handwork, etc.).  Don't know (DO NOT READ) 998  Don't Answer (DO NOT READ) 999 | Per Hectare   Total.   **\|__\|__\|__\|** € | Per Hectare   Total.   **\|__\|__\|__\|** € | Per Hectare   Total.   **\|__\|__\|__\|** € |

|  | **Year_D-Y** | | |
| --- | --- | --- | --- |
| [I.E.: ASK AS MANY APPLICATIONS AS INDICATED IN Q23] | **Application 1** | **Application 2** | **Application 3** |
| **Q39: Did this application consist of insecticides, fungicides and/or herbicides? [I.P.NOT FOR OSR]**  Multi coded. I.P.: ASK Q40A, B AND/OR C WHEN APPROPIATE  Yes, Herbicides (ASK Q40A HERBICIDES) 1  Yes, Insecticides (ASK Q40B INSECTICIDES) 2  Yes, Fungicides (ASK Q40C FUNGICIDES) 3 | 1   2   3  | 1   2   3  | 1   2   3  |
| **Q40: Which of the following products (active substances) were contained in this application? Please indicate the active substance and the brand name belonging to the active substance. If you do not know the active substance you used, please indicate at least the brand name.**  [READ OUT depending on the answer given in Q25; All the OPTIONS included] Multi coded. SHOWCARD  A. HERBICIDES [I.P.NOT FOR OSR]  Glyphosate 1  Terbuthylazine 2  Other herbicide (specify) 97  Don't know (DO NOT READ) 98 Don't Answer (DO NOT READ) 99  **B. INSECTICIDES**  MAIZE  Abamectine 3  Ethoprophos 4  Alfa cypermethrin 5  Cyfluthrin 6  Hexythiazox 7  Chlorpyrifos 8  Chlorpyrifos Methyl 9  Deltamethrin 10  Lambda Cyhalothrin 11  Tefluthrin 12  SUNFLOWER  Deltamethrin 13  Chlorpyrifos 14  Pirimicarb 15  Teflutrhin 16  OSR  Cypermethrin 17  Deltamethrin 18  Esfenvalerate 19  Lambda Cyhalothrin 20  Pirimicarb 21  Other insecticide (specify) 91  Don't know active substance (DO NOT READ) 92  Don't Answer (DO NOT READ) 93  **C. FUNGICIDES [I.P.NOT FOR OSR]**  **MAIZE**  Cyproconazole Epoxiconazole 22  Sulphur 23  Mancozeb 24  Methyl Thiphanate 25  **SUNFLOWER**  Fenpropimorph 26  Mancozeb 27  **OSR**  Mancozeb 27  Methyl Thiphanate 29  Propiconazole 30  Tebuconazole 31  Other fungicide (specify) 94  Don't know (DO NOT READ) 95  Don't Answer (DO NOT READ) 96 | 1 ______  2 ______  97 ______  98   99   3 ______  4 ______  5 ______  6 ______  7 ______  8 ______  9 ______  10 ______  11 ______  12 ______  13 ______  14 ______  15 ______  16 ______  17 ______  18 ______  19 ______  20 ______  21 ______  91 ______  92   93   22 ______  23 ______  24 ______  25 ______  26 ______  27 ______  27 ______  29 ______  30 ______  31 ______  94 ______  95   96  | 1 ______  2 ______  97 ______  98   99   3 ______  4 ______  5 ______  6 ______  7 ______  8 ______  9 ______  10 ______  11 ______  12 ______  13 ______  14 ______  15 ______  16 ______  17 ______  18 ______  19 ______  20 ______  21 ______  91 ______  92   93   22 ______  23 ______  24 ______  25 ______  26 ______  27 ______  27 ______  29 ______  30 ______  31 ______  94 ______  95   96  | 1 ______  2 ______  97 ______  98   99   3 ______  4 ______  5 ______  6 ______  7 ______  8 ______  9 ______  10 ______  11 ______  12 ______  13 ______  14 ______  15 ______  16 ______  17 ______  18 ______  19 ______  20 ______  21 ______  91 ______  92   93   22 ______  23 ______  24 ______  25 ______  26 ______  27 ______  27 ______  29 ______  30 ______  31 ______  94 ______  95   96  |
| **Q41: What type of treatment did you use in this application?**  Single coded  Soil treatment 1  Foliar treatment 2  Other (specify) 4  Don't know (DO NOT READ) 8  Don't Answer (DO NOT READ) 9 | 1   2   4   8   9  | 1   2   4   8   9  | 1   2   4   8   9  |
| **Q42: What was the crop stage when the application took place?** READ OUT, single code  Before Sowing 1  During Sowing 2  After sowing/preemergency 3  Vegetative stage 4  Flowering stage 5  Fructification stage 6  Other (DO NOT READ) 8  Don't Answer (DO NOT READ) 9 | 1   2   4   5   6   8   9  | 1   2   4   5   6   8   9  | 1   2   4   5   6   8   9  |
| **Q43: What was the pest treated with this application?**  (I.E.: SHOWCARD AND READ OUT LOUD. Multi coded)  (I.P.: IF herbicide code 1, “Weeds”, should automatically be assigned)  (I.E.: SHOWCARD AND READ OUT LOUD. Multi coded.**I.P.: IF herbicide code 1, “Weeds”, should automatically be assigned)**  [I.P. OSR would not be asked about weeds].  (MAIZE cod.2-7, & 27)  Corn Borer (ECB/WCB) 2  Cutworms 3  Wireworms 4  Lepidopteran leaf feeders 5  Leafhoppers 6  Insect borne viruses 7  Diabrotica virgifera 27  (OSR cod. 4-26; skip 5-7 & 21)  Wireworms 4  Alternaria 8  Peach/Potate Aphid 9  Brassica pod midge 10  Cabagge aphid 11  Cabagge flea beetle 12  Cabagge root fly 13  Cabagge seed weevil 14  Cabagge stem flea beetle 15  Cabagge stem weevil 16  Cylindrosporium 17  Pod midge 20  Pollen beetles 22  Rape stem weevil 23  Rape winter stem weevil 24  Sclerotinia 25  Phoma stem canker 27  (SUNFLOWER cod.3-39 skip 5-27)  Aphids 41  Cutworms 3  False wireworms 28  Heliothis / Helicoverpa spp. 29  Root and stem charcoal rot 30  Sunflower broomrape 31  Sunflower downy mildew 32  Sunflower moth 33  Verticilosis 34  Weevils, 35  White grubs, 36  White rot 37  Wireworms 4  Gray rot 38  Sunflower rust 39  Grey Spot 40  Other (specify) 97  Don't know (DO NOT READ) 98  Don't answer (DO NOT READ) 99 | 2   3   4   5   6   7   27   4   8   9   10   11   12   13   14   15   16   17   20   22   23   24   25   27   41   3   28   29   30   31   32   33   34   35   36   37   4   38   39   40   97 _______  98   99  | 2   3   4   5   6   7   27   4   8   9   10   11   12   13   14   15   16   17   20   22   23   24   25   27   41   3   28   29   30   31   32   33   34   35   36   37   4   38   39   40   97 _______  98   99  | 2   3   4   5   6   7   27   4   8   9   10   11   12   13   14   15   16   17   20   22   23   24   25   27   41   3   28   29   30   31   32   33   34   35   36   37   4   38   39   40   97 _______  98   99  |
| **Q44: What was the cost of the treatment?**  (I.P.; control currency unit)  (I.E.: Cost of application including product and machinery, handwork, etc.).  Don't know (DO NOT READ) 998  Don't Answer (DO NOT READ) 999 | Per Hectare   Total.   **\|__\|__\|__\|** € | Per Hectare   Total.   **\|__\|__\|__\|** € | Per Hectare   Total.   **\|__\|__\|__\|** € |

**Q45: [skip if Q7 = 2]. Are you aware of the recent EU regulations restricting the use of**

**(I.P.: Include the list of products for the crop/country, used in question 11) ("neonicotinoids"/ "Fipronil") or seed, soil and foliar treatments of [MAIZE/OSR/SUNFLOWER]?**

Multi coded (READ OUT)

Yes. 1 

Q45a. If Yes; What have you heard about it?_____________________

No 2 

Q45b. If No: (READ OUT) In the EU, the use of some neonicotinoids (I.P.: Include the list of Neonicotinoids products for the crop/country, used in question 11) for seed, soil and foliar (before flowering) treatments is banned since December 2012. Also, the use of Fipronil (I.P.: Include the list of Fipronil products for the crop/country, used in question 11) for seed treatments is banned since March 2013.

Don't Answer (DO NOT READ) 9 

**Q46: [skip if Q7 = 2]. Have you changed any of your crop protection practices in [MAIZE/OSR/SUNFLOWER] in Year_D as compared to Year_D-X because of the restrictions of the use of (I.P.: Include the list of products for the crop/country, used in question 11)?** Single coded (DO NOT READ)

Yes 1 

No 2 

Don't know 8 

Don't Answer 9 

**Q47: [skip if Q7 = 2]. After the restrictions of the use of (I.P.: Include the list of brands for the crop/country, used in question 11) which measures, if any, have you undertaken in [MAIZE/OSR/SUNFLOWER]?** Multi coded, (READ OUT)

Increase sowing density 1 

Earlier sowing date 2 

Later sowing date 3 

Reduce area of [MAIZE/OSR/SUNFLOWER] 4  [Do Q54]

Use alternative seed insecticide treatments 5 

Use more soil insecticide treatments 6 

Use more foliar insecticide treatments 7 

Use more mechanical pest control practices 8 

More frequent scouting for pests 9 

Other (specify) ________________________ 10 

Don't know (DO NOT READ) 11 

**Q48:** **[skip if Q7 = 2]. Do you think that overall, due to the restrictions of (I.P.: Include the list of products for the crop/country, used in question 11), protecting your [MAIZE/OSR/SUNFLOWER] is becoming more time-consuming, less time-consuming, or staying similarly time-consuming as compared to before?**

Single coded

More time-consuming 1 

Less time-consuming 2 

Similar 3 

Don't Know (DO NOT READ) 8 

Don't Answer (DO NOT READ) 9 

**Q49:** **[skip if Q7 = 2]. Do you think that overall, due to the restrictions of (I.P.: Include the list of products for the crop/country, used in question 11) protecting your [MAIZE/OSR/SUNFLOWER] is becoming more expensive, less expensive or staying similarly expensive as compared to before?**

Single coded (DO NOT READ)

More expensive 1 

Less expensive 2 

Similar 3 

Don't know (DO NOT READ) 98 

**Q50:** **[skip if Q7 = 2]. Do you think that overall, due to the restrictions of (I.P.: Include the list of products for the crop/country, used in question 11) protecting your [MAIZE/OSR/SUNFLOWER] is requiring more, fewer, or a similar amount of chemical plant protection products as compared to before?**

More 1 

Less 2 

Similar 3 

Don't know (DO NOT READ) 8 

**Q50a.** **[skip if Q7 = 2].** [I.P.: only ask if farmer switched from using a restricted seed treatment product before the restrictions to using a non-restricted seed treatment product after the restrictions indicated by his answer to Q12]

**You have switched from using (I.P.: name those seed treatment product(s) the farmer used in D-X or D-Y), to using (I.P.: name those seed treatment product(s) the farmer used in D, as indicated in Q12) with your [MAIZE/OSR/SUNFLOWER] seeds. How do you compare the overall effectiveness of these products as part of your pest management practices in [MAIZE/OSR/SUNFLOWER]?**

[I.E.: READ OUT / I.P.: MULTIPLE ANSWERS]

Product(s) used in D-X (D-Y) more effective 1 

Product(s) used in D-X (D-Y) somewhat more effective 2 

Products equally effective 3 

Product(s) used in D somewhat more effective 4 

Product(s) used in D more effective 5 

Don’t know (DO NOT READ) 8 

Don’t Answer (DO NOT READ) 9 

**Q51: [skip if Q7 = 2]. In [MAIZE/OSR/SUNFLOWER], but also in general on your farm, have you noticed higher or lower pressure from SOIL PESTS in Year_D as compared to Year_D-X?**

Multi coded (READ OUT)

Higher incidence. 1 

Lower incidence 2 

Similar incidence 2 

Q51a. If higher or lower; Which ones have a higher/lower incidence? _____________________

**Q52: [skip if Q7 = 2]. In [MAIZE/OSR/SUNFLOWER], but also in general on your farm, have you noticed higher or lower pressure of FOLIAR PESTS in Year_D as compared to Year_D-X?**

Multi coded (READ OUT)

Higher incidence. 1 

Lower incidence 2 

Similar incidence 2 

Q52a. If higher or lower; Which ones have a higher/lower incidence? _____________________

**Q53: [skip if Q7 = 2]. In [MAIZE/OSR/SUNFLOWER], but also in general on your farm, have you noticed higher or lower incidence of WILD BENEFICIAL INSECTS (e.g. pollinators, predators of pests) in Year_D as compared to Year_D-1?**.

Multi coded (READ OUT)

Higher incidence. 1 

Lower incidence 2 

Similar incidence 2 

Q53a. If higher or lower; Which ones have a higher/lower incidence? _____________________

**Q54. Have you grown any substitute crops on the area of [MAIZE/OSR/SUNFLOWER] that you reduced because of the restrictions of (I.P.: Include the list of products for the crop/country, used in question 11)? Please indicate all the different crops (including unused land) along with their area and estimated gross margins you have realized with them.**

**I.E.: Ask this question only to those farmers who indicated in Q47 "Reduce area of [MAIZE/OSR/SUNFLOWER]". For those who did not indicate that, skip this question.**

| **[substitute crop]** | ***Hectares*** | ***Gross margin*** |  |
| --- | --- | --- | --- |
| \|__________________\| | \|___\|___\|___\|___\| **ha** | \|___\|___\|___\|___\| **€** |  PER HECTARE   TOTAL |
| \|__________________\| | \|___\|___\|___\|___\| **ha** | \|___\|___\|___\|___\| **€** |  PER HECTARE   TOTAL |
| \|__________________\| | \|___\|___\|___\|___\| **ha** | \|___\|___\|___\|___\| **€** |  PER HECTARE   TOTAL |
| **Unused** | \|___\|___\|___\|___\| **ha** | \|___\|___\|___\|___\| **€** |  PER HECTARE   TOTAL |

**Q55. Imagine the restrictions of (I.P.: Include the list of products for the crop/country, used in question 11) would be removed and the products become again available for use in [MAIZE/OSR/SUNFLOWER]: Considering all monetary and non-monetary factors, how much value would this give to you? Please indicate the value per hectare.**

0€ 1 

1-5€ 2 

6-10€ 3 

11-15€ 4 

16-20€ 5 

21-25€ 6 

More than 25€ 7 

Don't Know (DO NOT READ) 8 

Don't Answer (DO NOT READ) 9 

**Q56. [I.E./I.P.: Ask only if Q55>1< 8]**

Finally in this case, could you please tell me what characteristics you value most about (I.P.: Include the list of products for the crop/country, used in question 11)?

Easy to use 1 

Low price / cost 2 

Easy to get 3 

Effectiveness 4 

Don’t need to use other treatments additionally 5 

Other (Specify)_________________ ________________________________ 6 

Don't Know (DO NOT READ) 8 

Don't Answer (DO NOT READ) 9 

| **B4: DEMOGRAPHICS OF THE RESPONDENT** |
| --- |

**D1: What is the ownership status of the farm?**

(READ OUT).Single coded

Individual/Family farm household 1 

Private corporation 2 

Public company 3 

Other (Specify)_________________7 

Don't know (DO NOT READ) 8 

Don't Answer (DO NOT READ) 9 

**D2: Are you a member of any association or cooperative of farmers?**

(READ OUT). Rotate items 1-5. Multi coded

Cooperative 1 

Agrarian society (not in France) 2 

Farmers Association 3 

Union 5 

Other 6 

None of the above 7 

Don't know (DO NOT READ) 8 

Don't Answer (DO NOT READ) 9 

**D3: How old is the head of the farm/manager?**

Min 18 | Max 100 |___|___|___|

**D4: What is the gender of the head of the farm/manager?**

Ask only if necessary. Single coded

Man 1 

Woman 2 

**D5: What is the head of the farm/manager level of education?**

Single coded

Primary 1 

Secondary 2 

University 3 

Don’t know (DO NOT READ) 8 

Don’t Answer (DO NOT READ) 9 

**D6: Does the head of the farm/manager have a specific education in agriculture?**

Single coded

Yes 1 

No 2 

Don’t know (DO NOT READ) 8 

Don’t Answer (DO NOT READ) 9 

**D7: In Year_D, which percentage of your income came from the farm and which from non-farming activities?**

**Please state the part for each of the two options. Total must add up to 100%**

|  |  |  | Don't Know (DO NOT READ) | Don't Answer (DO NOT READ) |
| --- | --- | --- | --- | --- |
| 1 | The farm | **\|___\|___\|___\|%** |  |  |
| 2 | Other activities | **\|___\|___\|___\|%** |  |  |

**D8: Which percentage of your farm income comes from [MAIZE/OSR/SUNFLOWER] production?**

| [MAIZE/OSR/SUNFLOWER] |  | Don't Know (DO NOT READ) | Don't Answer (DO NOT READ) |
| --- | --- | --- | --- |
| production | **\|___\|___\|%** |  |  |

**D10: And could you please tell us what is approximately your annual FARM GROSS INCOME, IN 2014?**

[SHOW CARD] Single coded

Below 15.000 € 1 

15.001 to 25.000 € 2 

25.001 to 50.000 € 3 

50.001 to 75.000 € 4 

75.001 to 100.000 € 5 

100.001 to 150.000 € 6 

150.0001 to 200.000 € 7 

200.001 to 300.000 € 8 

More than 300.000 € 9 

Don’t know (DO NOT READ) 98 

Don’t Answer (DO NOT READ) 99 

**Time end: l__l__l h l__l__l mn**

Thank you very much for your help.

WHEN SCREENED OUT: Unfortunately your farm characteristics do not meet our recruitment criteria.

| **B5: INTERVIEW QUALITY CONTROL** |
| --- |

**I1: Where did the interview took place?**

Single coded

At the house door of the interviewee 1 

In the house of the interviewee 2 

In the cooperative 3 

On the street or in a plot of the interviewee 4 

Other (Specify) ___________________________________________6 

**I2: Where other persons present at the moment of the interview?**

Yes 1 

No 2 

**I3: How was the comprehension level of the interviewee at the questions?**

Single coded

Very good 5 

Good 4 

Normal 3 

Bad 2 

Very bad 1 

Other (Specify)_____________________________________________6 

**I4: Which of the questions were more difficult to understand to the interviewee?**

**(Please indicate the number)**

|  |
| --- |

**I5: Please insert any comment of the interview that need to be taken into account: clarification to questions, etc.**

|  |
| --- |

[CONTACT DATA: NAME, ADRESSE, TELEPHONE NUMBER].
